# ALKBH1‐mediated m^1^A demethylation of *METTL3* mRNA promotes the metastasis of colorectal cancer by downregulating SMAD7 expression

**DOI:** 10.1002/1878-0261.13366

**Published:** 2022-12-30

**Authors:** Wenwen Chen, Hao Wang, Shuyi Mi, Liming Shao, Zhipeng Xu, Meng Xue

**Affiliations:** ^1^ Department of Gastroenterology The Second Affiliated Hospital of Zhejiang University School of Medicine Hangzhou China; ^2^ Institute of Gastroenterology Zhejiang University Hangzhou China

**Keywords:** ALKBH1, colorectal cancer metastasis, m^1^A modification, m^6^A modification, METTL3

## Abstract

Colorectal cancer (CRC) is one of the most common malignancies, and the main cause of death from CRC is tumor metastasis. m^1^A RNA modification plays critical role in many biological processes. However, the role of m^1^A modification in CRC remains unclear. Here, we find that the m^1^A demethylase alkB homolog 1, histone H2A dioxygenase (ALKBH1) is overexpressed in CRC and is associated with metastasis and poor prognosis. Upregulation of *ALKBH1* expression promotes CRC metastasis *in vitro* and *in vivo*. Mechanistically, knockdown of *ALKBH1* results in a decrease in methyltransferase 3, N6‐adenosine‐methyltransferase complex catalytic subunit (*METTL3*) expression, probably due to m^1^A modification of *METTL3* mRNA, followed by m^6^A demethylation of SMAD family member 7 (*SMAD7*) mRNA. In addition, downregulation of *SMAD7* establishes an aggressive phenotype. More importantly, the cell migration and invasion defects caused by ALKBH1 depletion or METTL3 depletion are significantly reversed by *SMAD7* silencing. Considering these results collectively, we propose that ALKBH1 promotes CRC metastasis by destabilizing SMAD7 through *METTL3*.

## Graphical abstract

Colorectal cancer (CRC) is one of the most common malignancies, and the main cause of death from CRC is tumor metastasis. Here, we identified the important role of ALKBH1‐catalyzed m^1^A modification in CRC metastasis. ALKBH1‐mediated m^1^A demethylation of *METTL3* mRNA promotes the metastasis of colorectal cancer by downregulating SMAD7 expression.

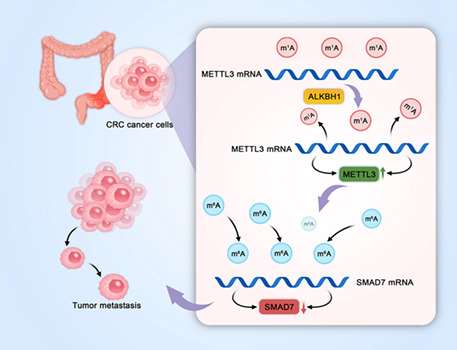

**Abbreviations**
AJCC: American Joint Committee on Cancer
ALKBH1: alkB homolog 1, histone H2A dioxygenase
ATF4: transcription factor 4
CHX: Cycloheximide
COAD: colon adenocarcinoma
CRC: colorectal cancer
FBS: fetal bovine serum
GEO: Gene Expression Omnibus
GEPIA: Gene Expression Profiling Interactive Analysis
GTEx: the Genotype‐Tissue Expression
H&E: hematoxylin and eosin
H3K27ac: histone H3 acetylation at lysine 27
IHC: immunohistochemical
KEGG: Kyoto Encyclopedia of Genes and Genomes
KM‐plotter: Kaplan–Meier Plotter
MeRIP: methylated RNA immunoprecipitation
METTL3: methyltransferase 3, N6‐adenosine‐methyltransferase complex catalytic subunit
OS: overall survival
READ: rectum adenocarcinoma
SD: standard deviations
SMAD7: SMAD family member 7
STR: short tandem repeat
TCGA: The Cancer Genome Atlas
TRMT6: tRNA methyltransferase 6
wt: wild type

## 1 Introduction

Colorectal cancer (CRC) is a malignant tumor that seriously threatens human health worldwide, with the third highest incidence rate and the second highest mortality rate [1]. At present, although great advances have been achieved in CRC from bench to bedside, the 5‐year survival rate of patients with metastatic CRC is still very low [2]. Therefore, understanding the molecular mechanisms of metastasis in CRC is essential for diagnostic and therapeutic inventions.

RNA methylation, a prevalent posttranscriptional modification, has been identified as a critical regulator of various biological processes, such as RNA transcription, splicing, structure, and stability, as well as protein translation [3, 4]. m^6^A is the most abundant modification in eukaryotic mRNAs and contributes significantly to the metastasis of cancer [5]. The presence of m^1^A modifications on ribonucleic acid was reported as early as 1961 [6]. However, the ratio of m^1^A/A is approximately 0.02% in mammalian cells and up to 0.16% in mammalian tissues, which is relatively low compared with the ratio of m^6^A/A [3, 7]. Recent studies have shown that m^1^A is present in rRNA, tRNA, mRNA, and mitochondrial RNA, and is a reversible and dynamic modification regulated by specific methyltransferases (writers) and demethylases (erasers) [7, 8, 9, 10, 11, 12]. The well‐characterized methyltransferases are tRNA methyltransferase 6 (TRMT6), TRMT61A, TRMT61B, and TRMT10C [3]. AlkB homolog 1, histone H2A dioxygenase (ALKBH1), and ALKBH3, which belong to the human AlkB family dioxygenases, have been reported to demethylate m^1^A in RNAs [3, 11]. In addition, recent studies suggest that YTHDF1, YTHDF2, YTHDF3, and YTHDC1 are putative m^1^A‐binding proteins functioning as readers [13].

The methyl group of m^1^A is located at the Watson‐Crick base pairing interface [10]. Accumulating studies indicate that m^1^A plays an important role in biological processes. In addition to the methylated group, the m^1^A‐modified nitrogen atom also has a positive charge and thus has a more powerful effect on the mRNA secondary/tertiary structure and RNA–protein interactions than the m^6^A‐modified one [7]. More importantly, defects in m^1^A methylation are associated with a range of human diseases, such as HIV, pulmonary hypertension, cardiovascular diseases, and cancer [14, 15, 16, 17, 18]. However, the role of m^1^A methylation in CRC, especially the invasiveness of CRC remains largely unknown. In this study, we reveal the biological role of m^1^A modification mediated by ALKBH1 and the downstream METTL3‐SMAD7 regulatory axis in CRC.

## 2 Materials and methods

### 2.1 Patients and samples

Tumor tissues embedded in paraffin were collected from 124 CRC patients undergoing radical surgery in the Second Affiliated Hospital of Zhejiang University School of Medicine, and paired adjacent normal tissues (> 5 cm away from tumor) were obtained from 116 CRC patients. A total of 124 CRC patients' clinical data, including sex, age, TNM stage, and pathological grade, were also collected. Follow‐up was performed successfully in 94 patients. In addition, we collected 20 other CRC tissue samples and performed immunohistochemical (IHC) analysis. No patient had received local or systemic treatment before the operation. The experiments were undertaken with the understanding and written consent of each subject. The study methodologies conformed to the standards set by the Declaration of Helsinki. Our study was approved by the Ethics Committee of the Second Affiliated Hospital of Zhejiang University School of Medicine (2022/0145).

### 2.2 Cell culture, transfections, and drug treatment

Human CRC cell lines HCT116 (RRID:CVCL_0291), HT29 (RRID:CVCL_0320), LoVo (RRID:CVCL_0399), RKO (RRID:CVCL_0504), SW620 (RRID:CVCL_0547), and SW480 (RRID:CVCL_0546) cells were maintained in our laboratory. All cell lines were obtained from the American Type Culture Collection (Manassas, VA, USA). All cell lines were routinely tested using a PCR‐based method and had negative results for mycoplasma. In addition, all the cell lines were authenticated by short tandem repeat (STR) profiling in the past 3 years before the experiments. Briefly, genomic DNA was extracted from cell line first, then primers for STR sequence were designed, followed by PCR amplification and electrophoresis detection. Finally, the STR typing results were compared with STR database.

HCT116 and HT29 cells were cultured in McCoy's 5A medium (Cienry, Huzhou, China), and LoVo cells were cultured in Ham's F‐12K (Kaighn's) medium (Thermo Fisher, Shanghai, China). RKO cells were maintained in Minimum Essential medium (Cienry). SW480 cells were cultured in RPMI‐1640 medium (Thermo Fisher). SW620 cells were cultured in Leibovitz's L‐15 medium (Thermo Fisher). All media were supplemented with 10% fetal bovine serum (FBS; ExCell Bio, Taicang, China) at 37 °C in 5% CO_2_. Plasmid transfection was carried out using PolyJet transfection reagent (SignaGen Laboratories, Rockville, MD, USA), and the siRNA duplexes were transfected with Lipofectamine RNAiMAX (Invitrogen, Carlsbad, CA, USA). The transfection process was performed according to the manufacturer's instructions. For Cycloheximide (CHX) (MedChemExpress, Shanghai, China) chase analysis, 50 μg·mL^−1^ CHX was used for the indicated times. MG132 (MedChemExpress) was stored at −20 °C as a stock solution at 5 mm in DMSO, and was added to HCT116 or RKO cells at a concentration of 10 μm for 4 h.

### 2.3 Plasmids and siRNAs

The full‐length human *ALKBH1* and *METTL3* sequences cloned by RT–PCR using extracted RNA from 293T cells were inserted into the pCDH‐puro vector to construct the OE‐ALKBH1 and OE‐METTL3 overexpression plasmids, respectively. All constructs were confirmed by DNA sequencing.

The ALKBH1 overexpression plasmid with site‐directed mutagenesis was constructed using the Mut Express MultiS Fast Mutagenesis Kit V2 (Vazyme, Nanjing, China). The following pair of primers were used:

H231A/D233A_forward: GGAATCGCCGTAGCCAGATCTGAG,
H231A/D233A_reverse: CTCAGATCTGGCTACGGCGATTCC [11].

All siRNAs were synthesized by GenePharma (Shanghai, China). The sequences of the sense strands of the siRNA duplexes were as follows:

*ALKBH1*: 5′‐GUGAUCAAAUCUCAGCUAATT‐3′.
*METTL3*: 5′‐CTGCAAGTATGTTCACTATGA‐3′.
*SMAD7*: 5′‐AGGUCACCACCAUCCCCACTT‐3′.

### 2.4 Antibodies

An anti‐m^6^A antibody (rabbit, Synaptic Systems, Goettingen, Germany, 202003) was used at 1 μg·mL^−1^ for immunofluorescence, and an anti‐m^1^A antibody (mouse, MBL, Beijing, China, D345‐3) was used at 1 μg·mL^−1^ for immunofluorescence. For western blotting analysis, antibodies against ALKBH1 (rabbit, 1 : 1000, Abcam, Cambridge, UK, ab126596), ALKBH5 (rabbit, 1 : 1000, Proteintech, Wuhan, China, 16837‐1‐AP), FTO (rabbit, 1 : 1000, Proteintech, 27226‐1‐AP), GAPDH (rabbit, 1 : 1000, diagbio, Hangzhou, China, db106), IGF2BP1 (rabbit, 1 : 1000, CST, Danvers, MA, USA, 8482), KIAA1429 (rabbit, 1 : 1000, Proteintech, 25712‐1‐AP), METTL3 (rabbit, 1 : 1000, diagbio, db318), METTL14 (rabbit, 1 : 1000, Proteintech, 26158‐1‐AP), SMAD7 (rabbit, 1 : 1000, ABclonal, Wuhan, China, A12343), WTAP (mouse, 1 : 1000, Proteintech, 60188‐1‐Ig), YTHDC1 (rabbit, 1 : 1000, ABclonal, A7318), YTHDC2 (ABclonal, R27443), YTHDF1 (rabbit, 1 : 1000, ABclonal, R27444), YTHDF2 (rabbit, 1 : 1000, ABclonal, A15616), and YTHDF3 (rabbit, 1 : 1000, ABclonal, A8395) were utilized. Alexa Fluor 488‐ and 568‐conjugated anti‐rabbit and anti‐mouse IgG (1 : 200, Invitrogen) were used as the secondary antibodies for immunofluorescence analysis. HRP‐conjugated secondary antibodies (1 : 10 000, diagbio, db10002) were used for western blot analysis.

### 2.5 IHC analysis

Cores with the largest dimension measuring 1.5 mm were punched from nonnecrotic areas of matched tumor tissues and adjacent nontumor tissues. Tissue microarray slides containing 4 μm thick microarray sections were constructed using standard techniques (in collaboration with Shanghai Superchip Company, Ltd., Shanghai, China). Tissue sections following deparaffinization, rehydration, and antigen retrieval were conjugated with ALKBH1, METTL3, or SMAD7 antibody at 4 °C overnight. After incubation with secondary antibodies and development of diaminobenzidine, the scanned slides were reviewed by two independent pathologists. The protein expression of ALKBH1, METTL3, or SMAD7 was measured by multiplication of the positive staining percentage score (0: 0–5% positive cells; 1: 6–50% positive cells; 2: 51–75% positive cells and 3: 76–100% positive cells) and the staining intensity score (no staining = 0; weak staining = 1; moderate staining = 2; dense staining = 3). A score ≤ 3 was considered to indicate low expression, and a score ≥ 4 was considered to indicate high expression.

### 2.6 Western blot analysis

Whole‐cell extracts were obtained by lysis in TBSN buffer [20 mm Tris (pH 8.0), 150 mm NaCl, 0.5% Nonidet P‐40, 5 mm EGTA, 1.5 mm EDTA, 0.5 mm Na_3_VO_4_, and 20 mm p‐nitrophenyl phosphate] supplemented with protease inhibitors. Western blotting was performed with the indicated antibodies, and protein levels were developed using ECL Western Blot Substrate (NCM Biotech, Suzhou, China). Azure 600 Gel Imaging System, together with the azure capture software (1.6.3.1211, Azure Biosystems, Dublin, CA, USA) and azurespot (2.2.167) from Azure Biosystems were used to capture western blot bands.

### 2.7 Immunofluorescence staining

Cells were grown on glass coverslips, fixed for 15 min with 4% paraformaldehyde at room temperature and then incubated with primary antibodies for 2 h and secondary antibodies for 1 h at room temperature. DNA was stained with DAPI (Sigma‐Aldrich, St. Louis, MO, USA). The images were acquired using a 63× oil immersion objective.

### 2.8 Transwell assay

The migration potential was measured using a transwell apparatus (8‐μm pore size, Corning, NY, USA). A 200 μL volume of cell suspension containing 100 000 transfected cells was plated in medium containing 1% FBS in the top chamber of a transwell apparatus with or without coated matrigel (BD Bioscience, Franklin Lakes, NJ, USA), while 700 μL of medium containing 20% FBS was placed in the lower chamber. After 36 h of incubation, cells were fixed with 4% paraformaldehyde for 15 min and stained with 0.1% crystal violet for 20 min. Cells on the undersides of the filters were photographed using a microscope (magnification 200×). At least three random fields in each well were imaged and analyzed.

### 2.9 Wound healing assay

Wound healing assays were performed as described previously [19]. Briefly, transfected cells were seeded into 30 mm dishes with 10% serum‐containing culture medium. When the cells were confluent, they were starved for 12 h and scratched with a 20 μL pipette tip to create wounds. The cells were washed to remove debris and then replaced with 1% serum culture medium to allow wound healing. The cells were monitored by microscopy, and representative images were acquired at the indicated time points. The distance between the two boundary lines was measured by imagej software (NIH, Bethesda, MD, USA).

### 2.10 Quantitative real‐time RT–PCR

Quantitative RT‐PCR was performed using a LightCycler^®^ 480 II System (Roche, Basel, Switzerland) with HiScript Q RT SuperMix (Vazyme). All of the reactions were performed in triplicate. *GAPDH* was used as the internal control. The primers used to amplify the target regions were as follows:

*ALKBH1* Forward: AGAAGCGACTAAACGGAGACC
*ALKBH1* Reverse: GGGAAAGGTGTGTAATGATCTGC
*SMAD7* Forward: CCAACTGCAGACTGTCCAGA
*SMAD7* Reverse: TTCTCCTCCCAGTATGCCAC
*CSRNP1* Forward: GCCACAGCCTTTATTCCAG
*CSRNP1* Reverse: TTCCTGTTTCCTCCTTCCC
*TRIM15* Forward: GCCCGCTGGGAGAAACTTAC
*TRIM15* Reverse: GGCATCGTTCTCGCAGAAG
*RHOB* Forward: CGGACTCGCTGGAGAACA
*RHOB* Reverse: GAGGTAGTCGTAGGCTTGGAT
*GAPDH* Forward: ATTCCATGGCACCGTCAAGGCTGA
*GAPDH* Reverse: TTCTCCATGGTGGTGAAGACGCCA

### 2.11 Methylated RNA immunoprecipitation (MeRIP) qPCR assay

The m^6^A and m^1^A modifications of individual genes were determined using a MeRIP‐qPCR assay. Briefly, cells cultured in a 10 cm plate were washed twice with ice‐cold PBS and harvested by scraping in 1 mL of PBS. Total RNA was isolated using TRIzol. One‐tenth of the RNA was saved as the input control. A total of 40 μg of RNA and 2 μg of antibody were added to immunoprecipitation buffer supplemented with RNase inhibitors [20 mm Tris–HCl (pH 7.5), 140 mm NaCl, and 0.05% Triton X‐100] and incubated overnight at 4 °C with rotation. Then, prewashed Protein A/G magnetic beads were added and incubated for 4 h. Then, RNA was combined with the beads. After five washes, we eluted methylated mRNAs from the beads with TRIzol. The eluted RNAs were precipitated with ethanol and dissolved in RNase‐free water. Enrichment of certain fragments was determined by real‐time PCR. The proportion of m^6^A‐enriched RNA to input RNA is presented as the m^6^A level. The m^1^A enrichment in each sample was calculated by normalization to the input.

### 2.12 Cell proliferation and colony formation assays

Cell Counting Kit‐8 (CCK8, Meilunbio, Dalian, China) was used to test cell proliferation. The indicated cells were seeded in a 96‐well plate with 5000 cells per well. After different culture times, 10 μL of CCK‐8 reagent was added to each well and then cultured for 2 h. All experiments were performed in triplicate. The absorbance was analyzed at 450 nm on an automatic microplate reader (TECAN Spark^®^, Morrisville, NC, USA). The proliferation rate of cells was calculated. For colony formation assays, cells were placed in 6‐well plate with 3000 cells per well. After 5 days, cell colonies were stained with 0.1% of crystal violet and imaged. Each experiment was analyzed in triplicate.

### 2.13 FACS assay

The indicated cells were harvested and washed with PBS. The cells were mixed well with 500 μL PI binding buffer (MultiSciences Co., Hangzhou, China, CCS012) and incubated in dark for 30 min. Cell cycle was analyzed by flow cytometer (Beckman Coulter, Brea, CA, USA, CytoFLEXLX). For the cell apoptosis assay, the indicated cells were harvested and washed with PBS for Annexin V‐PI cell apoptosis assay (MultiSciences Co., AP101) using flow cytometry.

### 2.14 Animal experiments

All animal experiments were approved by the Ethics Committee of the Second Affiliated Hospital of Zhejiang University School of Medicine (2022/004). 4–5 weeks old female Balb/c athymic nude mice were purchased from Vital River (Beijing, China) and were maintained under SPF conditions with individually ventilated cages in the Animal Facility of the Second Affiliated Hospital of Zhejiang University School of Medicine. All mice were housed under a 12‐h light–dark cycle. A mouse model of lung metastasis was established by tail vein injection. When CRC cells were in the logarithmic growth phase in a 10 cm dish, they were harvested by trypsinization and were then resuspended and diluted to a concentration of 5 × 10^6^ tumor cells per 200 μL of solution. There were nine female nude mice aged 4–5 weeks in each group. The tail veins of the nude mice were first sterilized. An insulin needle was inserted 0.5–1 cm into the tail vein, 200 μL of the cell suspension was slowly injected, and the needle was then quickly withdrawn. After that, a medical‐grade absorbent cotton ball was used to stop the bleeding. Then, the mice were fed in an SPF environment continually. We supervise the welfare of the animals every day. In the presence of pain, stress, or suffering, mice were immediately euthanized by cervical dislocation upon isoflurane anesthesia. Eight weeks later, the mice were sacrificed, and the lungs were resected, photographed, and fixed with 4% paraformaldehyde. Finally, lung tissues were embedded in paraffin for hematoxylin and eosin (H&E) staining.

### 2.15 Statistical analysis

The differential expression of ALKBH1 between tumor tissue and nontumor tissue was determined by the Mann–Whitney *U* test. The chi‐square test or Fisher's exact test was used to analyze the relationships between clinicopathological features and ALKBH1 staining scores. Means and standard deviations (SD) were calculated for all quantitative experiments. A linear regression test was used to determine the correlation between ALKBH1, METTL3, and SMAD7 levels. Comparisons were analyzed by Two‐tailed Student's *t* test or One‐way ANOVA to determine the significance (graphpad prism 6, GraphPad Software, San Diego, CA, USA). Survival curves were generated using the Kaplan–Meier method and compared using the log‐rank test. Statistical significance was specified as *P < 0.05, **P < 0.01, or ***P < 0.001.

### 2.16 Bioinformatics analysis

GenomicScape is a web tool to easily visualize and analyze high‐throughput data. Publicly available datasets have been uploaded to help us to investigate gene/ncRNA expression profile. GenomicScape (http://genomicscape.com/) was established based on data obtained from Gene Expression Omnibus (GEO) [20]. In our manuscript, the dataset of 177 patients from the Moffitt Cancer Center [the dataset named Smith, Colon cancer 1 (Moffitt)] was used to detect the correlation between the overall survival (OS) of CRC patients and the expression of m^1^A regulators in the online bioinformatics tool GenomicScape.

Additionally, we also examined the expression levels of m^1^A regulators in colon adenocarcinoma (COAD) and rectum adenocarcinoma (READ) using the online bioinformatics tool Gene Expression Profiling Interactive Analysis (GEPIA) (http://gepia.cancer‐pku.cn/), based on tumor and normal samples from the Cancer Genome Atlas (TCGA) and the Genotype‐Tissue Expression (GTEx) databases [21].

Kaplan–Meier Plotter (KM‐plotter) (https://kmplot.com/analysis/), an online tool to correlate the gene expressions with prognosis in 21 tumor types and the sources of GEO, EGA, and TCGA databases, was used to analyze the correlation between the overall survival (OS) and the expression of *SMAD7* in CRC patients [22].

## 3 Results

### 3.1 Elevated ALKBH1 expression correlates with poor prognosis in patients with CRC

**Fig. 1:**
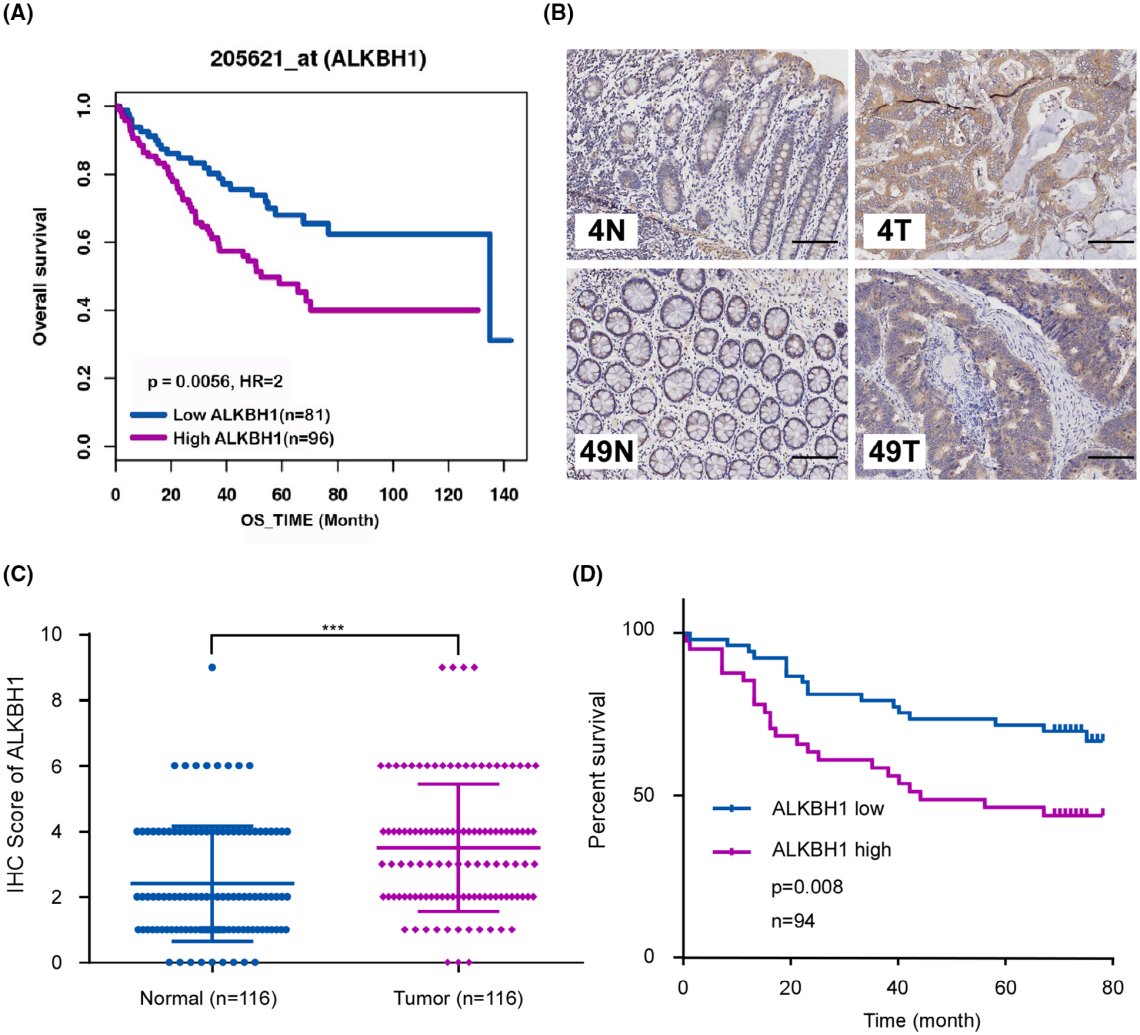
ALKBH1 is upregulated in colorectal cancer (CRC) tissues and related to the poor prognosis in patients. (A) Survival curve of overall survival (OS) based on *ALKBH1* expression using the online bioinformatics tool GenomicScape. Survival curves were generated using the Kaplan–Meier method and compared using the log‐rank test. (B) The expression levels of ALKBH1 were detected by immunochemistry analysis in 116 pairs of CRC tissues and adjacent noncancerous tissues. Representative images in 2 paired tumor (T) and normal (N) tissues were shown. Scale bar, 100 μm. (C) The comparison of ALKBH1 staining scores between CRC tissues (*n* = 116) and adjacent noncancerous tissues (*n* = 116). The error bars indicated mean with standard deviations (SD). Statistical analysis was performed using an unpaired two‐tailed Student's *t* test. (D) Survival curves were plotted based on the Kaplan–Meier survival analysis and compared using the log‐rank test. The expression level of ALKBH1 was used as the variate to separate two lines. ***P < 0.001. *n*, sample size.

In an attempt to elucidate the biological function of m^1^A modification in CRC, we first detected the correlation between the OS in CRC patients and the expression of m^1^A regulators using the online bioinformatics tool GenomicScape, including methyltransferases TRMT6, TRMT61A, TRMT61B and demethylases ALKBH1 and ALKBH3. The results showed that CRC patients with increased *ALKBH1* mRNA levels had worse overall survival, and the significance was strongest among five regulators (Fig. 1A, Fig. S1). Additionally, we also examined the expression levels of m^1^A regulators in COAD and READ using the online bioinformatics tool GEPIA, which showed that ALKBH1 expression was higher in tumor tissues than that in normal tissues (Fig. S2). To determine the expression pattern of ALKBH1 in CRC, 116 pairs of surgical specimens of human CRC tissues and adjacent noncancerous tissues were examined. The intensity of ALKBH1 staining was scored as 0 (negative), 1 (weak), 2 (moderate), or 3 (dense), as determined independently by two pathologists (representative staining images for each score were shown in Fig. S3). The results showed that the expression level of ALKBH1 in tumor tissues was significantly higher than that in adjacent nontumor tissues (*P* < 0.001, representative images are shown in Fig. 1B, statistical results are shown in Fig. 1C). Moreover, ALKBH1 expression was evaluated with regard to the clinicopathological characteristics of 124 patients. Upregulated ALKBH1 expression was significantly associated with lymph node metastasis (*P* = 0.0014), distant metastasis (*P* = 0.0091), and advanced AJCC stage (*P* = 0.0007; Table 1). More importantly, survival analysis of 94 patients using the Kaplan–Meier method indicated that CRC patients with high ALKBH1 expression exhibited worse overall survival (*P* = 0.008; Fig. 1D). Collectively, these results indicate that ALKBH1 is significantly upregulated in CRC and might be associated with metastasis.

**Table 1:** Relationship between the expression of ALKBH1 and clinicopathologic factors of colorectal cancer (CRC) patients.

Characteristics	Total (number)	ALKBH1 expression		
		Low (number)	High (number)	*P* value
Gender				
Male	69	35	34	0.6721
Female	55	30	25	
Age (years)				
≥ 65	63	30	33	0.2767
< 65	61	35	26	
T stage				
T1/2	24	15	9	0.2708
T3/4	100	50	50	
N stage				
N0	77	49	28	0.0014
N1/2	47	16	31	
M stage				
M0	112	63	49	0.0091
M1	12	2	10	
AJCC stage				
AJCC1/2	74	48	26	0.0007
AJCC3/4	50	17	33	

### 3.2 Depletion of ALKBH1 suppresses CRC cell migration and invasion

**Fig. 2:**
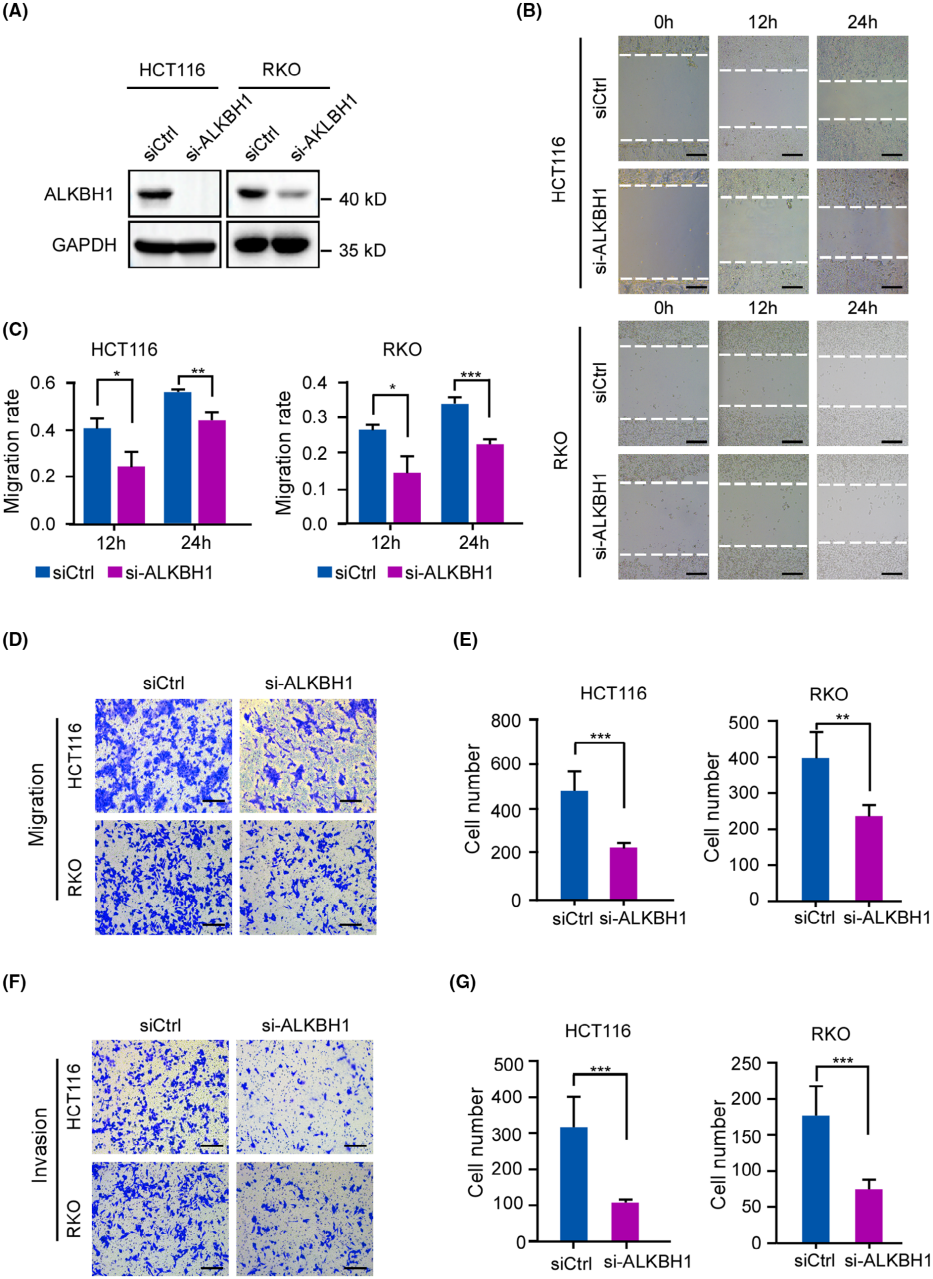
Knockdown of ALKBH1 suppresses colorectal cancer (CRC) cell migration and invasion. (A) HCT116 cells and RKO cells were transfected with control or ALKBH1 siRNAs for 48 h and then subjected to western blot analysis with anti‐ALKBH1 antibody. GAPDH was used as a loading control. Experiments were performed in triplicate. (B, C) The wound healing assays displayed cell migration at the different time points. Dashed lines indicate the wound edges. Scale bar, 200 μm. The distance between the two edge lines was measured by imagej software. Statistical analysis was performed using an unpaired two‐tailed Student's *t* test. Experiments were performed in triplicate. (D, E) Transwell migration assays revealed the cell migration ability of control and ALKBH1‐depleted cells. Scale bar, 200 μm. Cells that migrated to the undersides of the filters were counted. Statistical analysis was performed using an unpaired two‐tailed Student's *t* test. Experiments were performed in triplicate. (F, G) Transwell invasion assays were performed to detect the invasive ability of control and ALKBH1‐depleted cells. Scale bar, 200 μm. Cells that invaded to the undersides of the filters were counted. Statistical analysis was performed using an unpaired two‐tailed Student's *t* test. Quantitative data from three independent experiments are shown as the mean ± SD. *P < 0.05; **P < 0.01; ***P < 0.001.

To explore the crucial functions of ALKBH1 in CRC, we first examined ALKBH1 expression in CRC cell lines. Consistent with the results in CRC tissues, all 6 CRC cell lines were positive for ALKBH1 expression (Fig. S4A). In addition, we tested the migratory ability of CRC cells. The results showed that HCT116 and RKO cells migrated more readily (Fig. S4B). Thus, one siRNA oligo targeting *ALKBH1* mRNA was used in HCT116 and RKO cells, and the protein level of ALKBH1 was substantially reduced 72 h post‐transfection (Fig. 2A). The wound healing assay revealed that ALKBH1‐depleted cells climbed slower than control cells (Fig. 2B,C). In addition, we performed transwell migration and invasion assays *in vitro*. The data showed that downregulation of ALKBH1 in HCT116 and RKO cells inhibited cell penetration into the lower chamber compared to that of the corresponding control cells (Fig. 2D–G). Additionally, we also tested the cell cycle, proliferation, and apoptosis in ALKBH1‐depleted cells, which indicated that knockdown of ALKBH1 had no effect on the viability of CRC cells (Figs S5 and S6). Thus, these data suggest that ALKBH1 depletion suppresses CRC cell migration and invasion.

### 3.3 Exogenous ALKBH1 promotes CRC cell migration, invasion, and metastasis

**Fig. 3:**
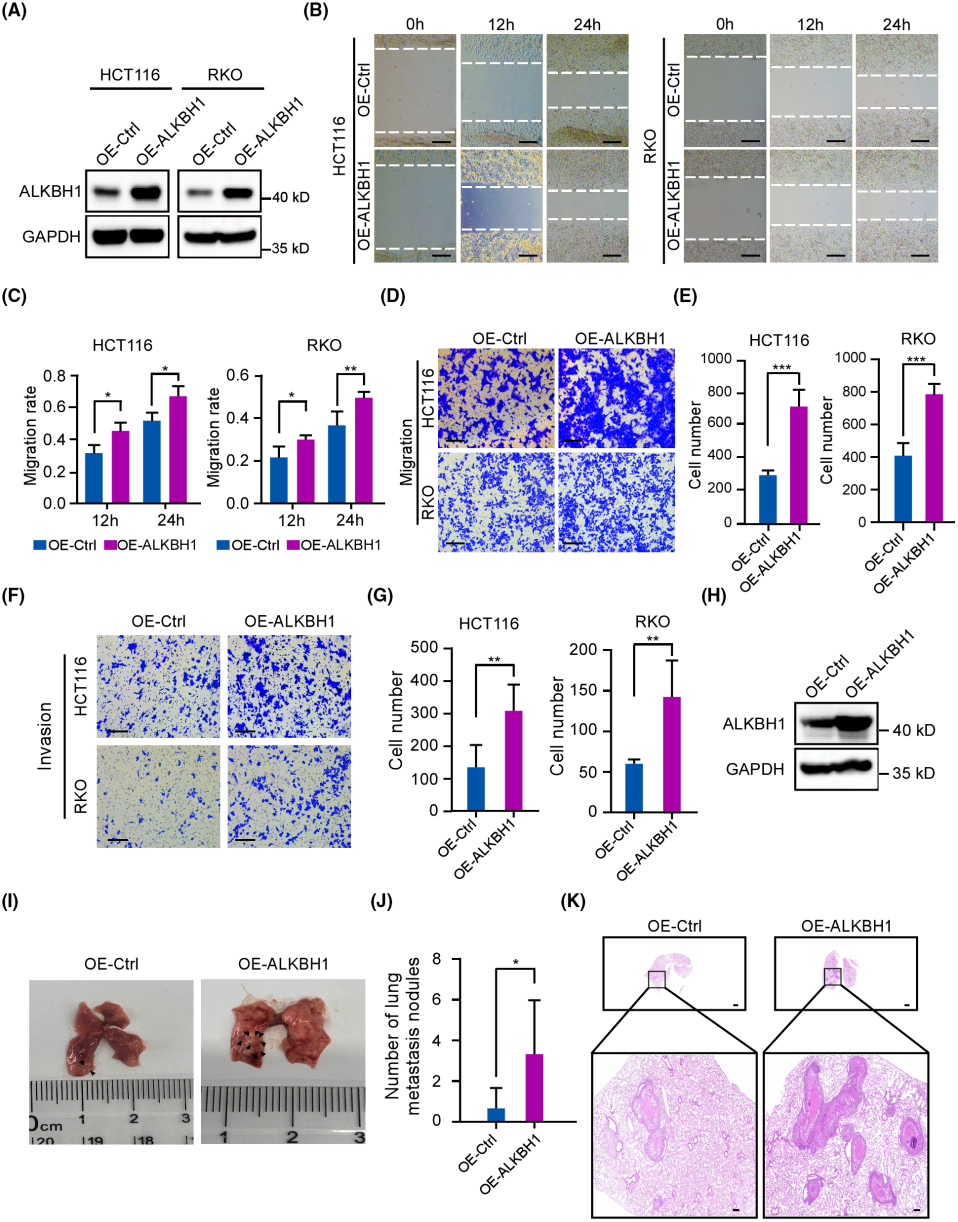
Overexpression of ALKBH1 promotes colorectal cancer (CRC) cell migration, invasion, and metastasis. (A) HCT116 cells and RKO cells transfected with control and ALKBH1 plasmids for 48 h were subjected to western blot analysis with anti‐ALKBH1 antibody. GAPDH was served as a loading control. Experiments were performed in triplicate. (B, C) The wound healing assays displayed cell migration at the different time points. Dashed lines indicate the wound edges. Scale bar, 200 μm. The distance between the two edge lines was measured by imagej software. Statistical analysis was performed using an unpaired two‐tailed Student's *t* test. Experiments were performed in triplicate. (D, E) Transwell migration assays revealed the cell migration ability of control and ALKBH1 overexpression cells. Scale bar, 200 μm. Cells that migrated to the undersides of the filters were counted. Statistical analysis was performed using an unpaired two‐tailed Student's *t* test. Experiments were performed in triplicate. (F, G) Transwell invasion assays were performed to detect the invasive ability of control and ALKBH1 overexpression cells. Scale bar, 200 μm. Cells that invaded to the undersides of the filters were counted. Statistical analysis was performed using an unpaired two‐tailed Student's *t* test. Experiments were performed in triplicate. (H) The protein levels of ALKBH1 in HCT116 cells with ALKBH1 overexpression were measured by western blot analysis. Experiments were performed in triplicate. (I, J) Representative images of the metastatic nodes in the lungs and quantification of the metastatic nodes (*n* = 6). Statistical analysis was performed using an unpaired two‐tailed Student's *t* test. (K) Hematoxylin and eosin (H&E)‐stained lung sections. Scale bar in the above picture is 1000 μm and scale bar in the down picture is 200 μm. Experiments were performed in triplicate. Quantitative data from three independent experiments are shown as the mean ± SD. *P < 0.05; **P < 0.01; ***P < 0.001. *n*, sample size.

To further confirm the function of ALKBH1 in CRC, wound healing assays and transwell assays were performed in ALKBH1‐overexpressing CRC cells. Overexpression of ALKBH1 was first verified by western blot analysis (Fig. 3A). As expected, the wound healing, transwell migration, and invasion assays showed that ectopic ALKBH1 expression facilitated cell migration and invasion (Fig. 3B–G). To test our *in vitro* findings, we first established HCT116 cells stably overexpressing ALKBH1 (Fig. 3H), which were then injected into nude mice via the tail vein. After 8 weeks, the lungs were harvested from the nude mice, and more lung metastatic lesions were found in the ALKBH1 overexpression group (Fig. 3I). We also determined the number of nodules, which was significantly higher in the ALKBH1 overexpression group than that in the control group (Fig. 3J). In addition, H&E staining confirmed that the number of lung metastatic lesions was increased with ALKBH1 upregulation (Fig. 3K). Taken together, our results strongly indicate that ALKBH1 drives CRC cell migration, invasion, and metastasis.

### 3.4 ALKBH1‐catalyzed demethylation is essential for CRC cell migration and invasion

**Fig. 4:**
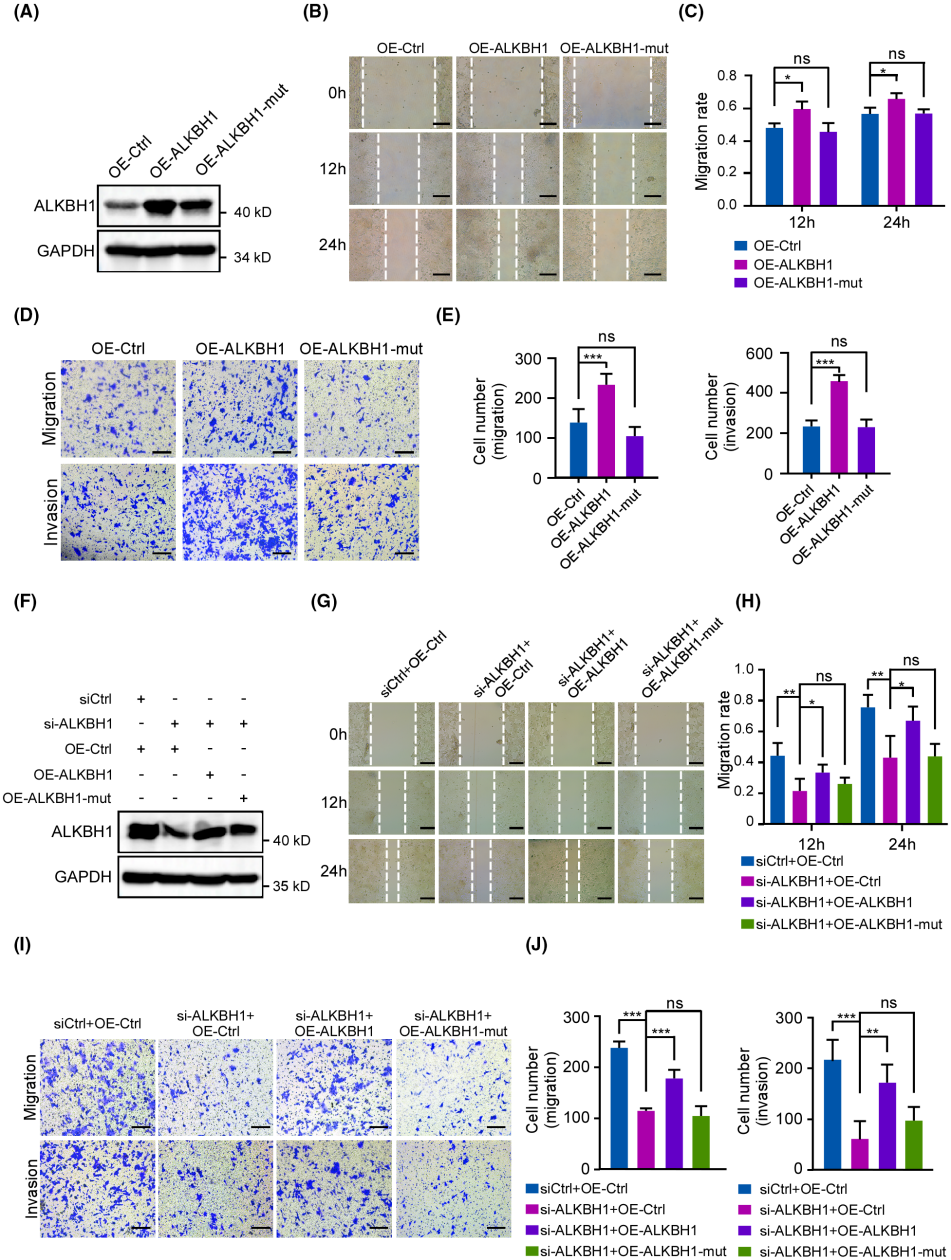
ALKBH1 accelerates colorectal cancer (CRC) cell migration and invasion through its m^1^A demethylation activity. (A) HCT116 cells transfected with control, ALKBH1, and mutant ALKBH1 plasmids for 48 h were subjected to western blot analysis with anti‐ALKBH1 antibody. GAPDH, a loading control. Experiments were performed in triplicate. (B, C) The wound healing assays revealed cell migration at the different time points. Dashed lines indicate the wound edges. Scale bar, 200 μm. The distance between the two edge lines was measured by imagej software. Comparisons were analyzed by one‐way ANOVA. Experiments were performed in triplicate. (D, E) Transwell migration assays and transwell invasion assays revealed the cell migration and invasion abilities. Scale bar, 200 μm. Cells that migrated to the undersides of the filters were counted. Comparisons were analyzed by one‐way ANOVA. Experiments were performed in triplicate. (F) HCT116 cells transfected with the indicated siRNAs and plasmids were subjected to western blot analysis with anti‐ALKBH1 antibody. GAPDH was used as a loading control. Experiments were performed in triplicate. (G, H) Wound healing assays detected cell migration at the different time points. The distance between the two edge lines was measured using imagej software. Scale bar, 200 μm. Comparisons were analyzed by one‐way ANOVA. Experiments were performed in triplicate. (I, J) Transwell migration and invasion assays were performed to detect cell migration and invasion. Scale bar, 200 μm. Cells that migrated to the undersides of the filters were counted. Experiments were performed in triplicate. Comparisons were analyzed by one‐way ANOVA. Quantitative data from three independent experiments are shown as the mean ± SD. *P < 0.05; **P < 0.01; ***P < 0.001; ns not significant.

Given that ALKBH1 mediates demethylation of m^1^A and that the ALKBH1 H231A/D233A mutation abolishes catalytic activity [11, 23], we generated an ALKBH1 H231A/D233A mutant construct to further determine whether ALKBH1 exerts its function through enzymatic activity. Wound healing and transwell assays showed that overexpression of mutant ALKBH1 had no significant effect on the migration and invasion of HCT116 cells (Fig. 4A–E). Moreover, ectopic expression of wild‐type ALKBH1 but not mutant ALKBH1 was able to reverse the defects in cell migration and invasion induced by ALKBH1 depletion (Fig. 4F–J). Similar results were also observed in RKO cells (Fig. S7). Collectively, these data imply that the m^1^A enzymatic activity of ALKBH1 is indispensable for its role in promoting CRC cell migration and invasion.

### 3.5 ALKBH1 affects METTL3 expression

**Fig. 5:**
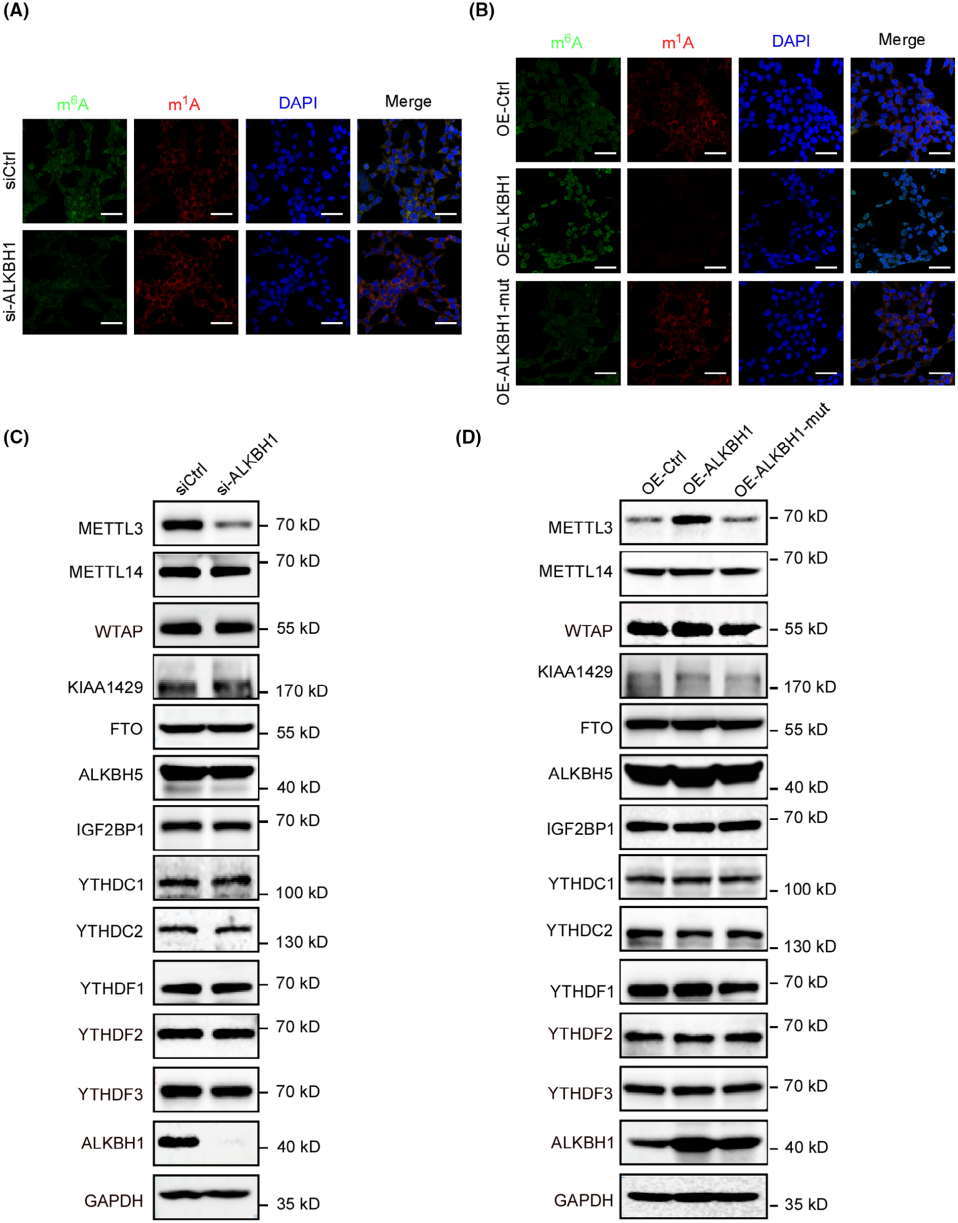
ALKBH1 affects METTL3 protein expression and METTL3‐mediated m^6^A modification. (A) HCT116 cells transfected with the indicated siRNAs were subjected to immunofluorescence. Cells were fixed and stained with anti‐m^6^A (green) and anti‐m^1^A (red). DNA was visualized with DAPI (blue). Scale bar, 30 μm. Experiments were performed in triplicate. (B) HCT116 cells transfected with the indicated plasmids were subjected to immunofluorescence. Cells were fixed and stained with anti‐m^6^A (green) and anti‐m^1^A (red). DNA was visualized with DAPI (blue). Scale bar, 30 μm. Three independent experiments were performed. (C, D) HCT116 cells transfected with the indicated siRNAs or plasmids were subjected to western blot analysis with the indicated antibodies. GAPDH was used as a loading control. Experiments were performed in triplicate.

Considering that m^1^A can be converted to m^6^A by Dimroth rearrangement and nitrogen atom exchange in an alkaline environment, and the alteration of ALKBH1 was accompanied by dysregulated m^6^A modification, as evidenced by a previous study in lung cancer [24, 25], we attempted to assess whether the m^6^A level was also altered when the m^1^A status was modified by ALKBH1 in CRC. Immunofluorescence assays using anti‐m^1^A and anti‐m^6^A antibodies showed that depletion of ALKBH1 simultaneously decreased m^6^A levels and increased m^1^A levels in HCT116 cells (Fig. 5A). Accordingly, overexpression of ALKBH1 led to the opposite result, while mutant ALKBH1 had no such effects (Fig. 5B). Furthermore, a similar phenomenon was found in RKO cells (Fig. S8A,B). To explore the underlying mechanisms by which ALKBH1 regulates m^6^A modification, we examined the protein levels of common m^6^A‐modified regulators in ALKBH1‐depleted HCT116 and RKO cells. Western blot analysis showed that only METTL3 protein levels were substantially decreased in ALKBH1‐depleted cells (Fig. 5C, Fig. S8C).

It is assumed that m^1^A is widespread in human mRNA and that AlkB can remove m^1^A methylation in mRNA. Moreover, ALKBH1 may mediate the demethylation of m^1^A in mRNA [11, 26]. To explore whether the role of ALKBH1 in regulating METTL3 expression is dependent on its role as a demethylase, we examined the protein levels of these proteins in ALKBH1‐overexpressing cells and mutant ALKBH1‐overexpressing cells. The data showed that METTL3 protein levels were increased only in ALKBH1‐overexpressing cells, not mutant ALKBH1‐overexpressing cells (Fig. 5D, Fig. S8D). It is worth noting that downregulation of ALKBH1 had no significant effect on the *METTL3* mRNA level (Fig. S9). What's more, we tested the METTL3 protein stability in ALKBH1‐depleted cells. CHX chase analysis revealed that the degradation rate of METTL3 was not altered in ALKBH1‐depleted HCT116 cells (Fig. S10A,B), implying that ALKBH1 is not involved in the regulation of METTL3 protein stability. Furthermore, we employed the proteasome inhibitor MG132 to treat ALKBH1‐depleted HCT16 cells and found that MG132 could not reverse the protein expression of METTL3 inhibited by *ALKBH1* siRNA (Fig. S10C). Similar results were also found in RKO cells (Fig. S10D–F). Collectively, these data suggest that ALKBH1 affects METTL3 protein expression and METTL3‐mediated m^6^A modification.

### 3.6 SMAD7 is a critical downstream target of ALKBH1

**Fig. 6:**
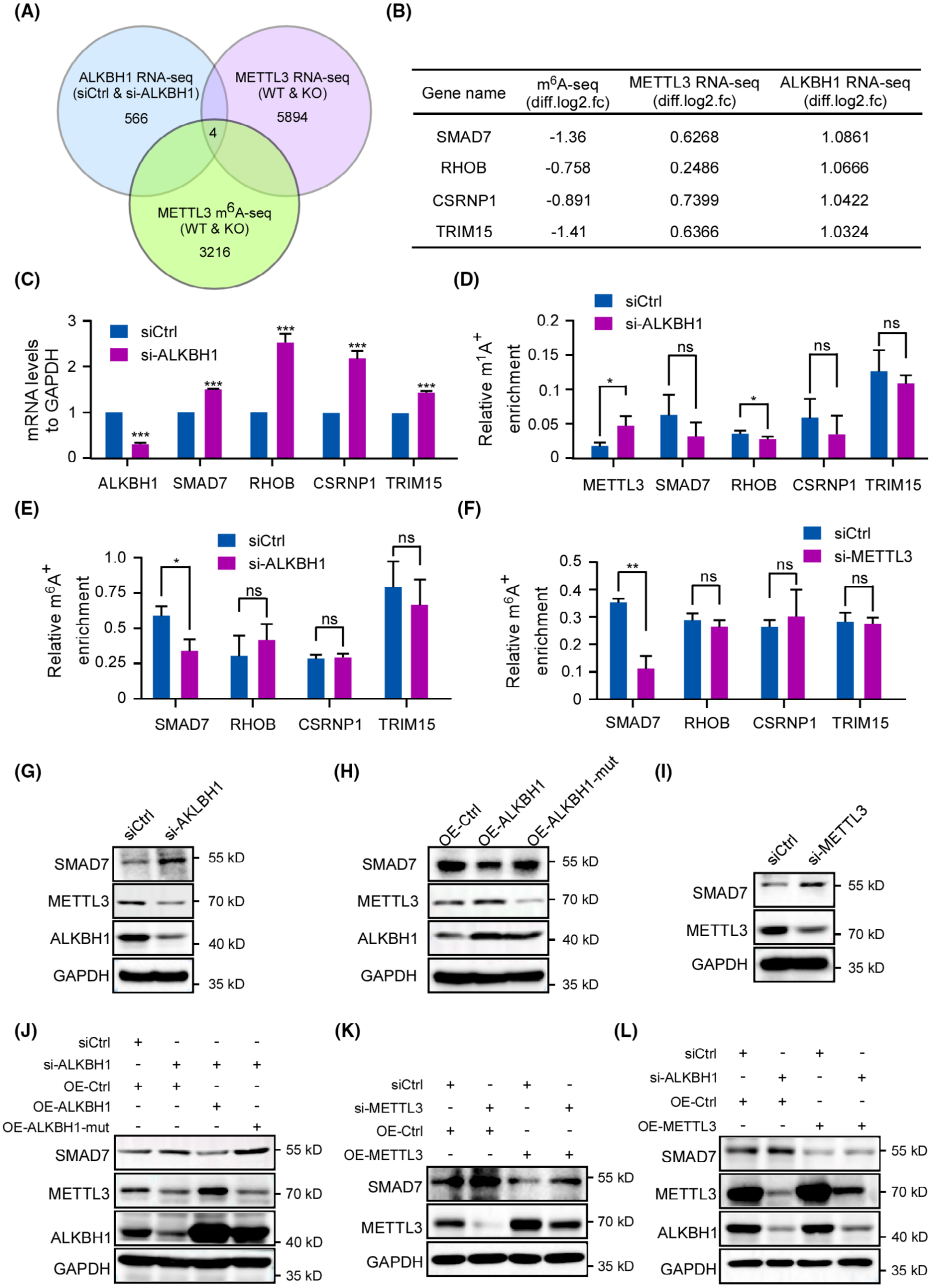
ALKBH1‐mediated m^1^A demethylation of *METTL3* mRNA inhibits SMAD7 expression by METTL3‐mediated m^6^A modification. (A) Venn diagram showed the overlap in ALKBH1 knockdown RNA‐seq dataset, METTL3 knockdown RNA‐seq dataset, and METTL3 knockdown m^6^A‐seq dataset. RNA‐seq was performed in triplicate in negative controls and ALKBH1‐depleted HCT116 cells. (B) Four overlapping genes were shown. (C) Quantitative real‐time RT‐PCR analysis of the indicated mRNA in control and ALKBH‐depleted HCT116 cells. GAPDH was used as an internal control. Experiments were performed in triplicate. (D) Methylated RNA immunoprecipitation (MeRIP)‐qPCR analysis of m^1^A level in the select mRNAs in control and ALKBH1‐depleted HCT116 cells. Experiments were performed in triplicate. (E) MeRIP‐qPCR analysis of m^6^A level in the select mRNAs in control and ALKBH1‐depleted HCT116 cells. Experiments were performed in triplicate. (F) MeRIP‐qPCR analysis of m^6^A level in the select mRNAs in control and METTL3‐depleted HCT116 cells. Experiments were performed in triplicate. (G–I) HCT116 cells transfected with the indicated individual siRNAs or plasmids were subjected to western blot analysis of the expression of SMAD7, METTL3, and ALKBH1. GAPDH was used as a loading control. Experiments were performed in triplicate. (J–L) HCT116 cells co‐transfected with the indicated siRNAs and vectors were subjected to western blot analysis with anti‐SMAD7, anti‐METTL3, and anti‐ALKBH1 antibodies. GAPDH was used as a loading control. Experiments were performed in triplicate. Quantitative data from three independent experiments are shown as the mean ± SD. *P < 0.05; **P < 0.01; ***P < 0.001; ns not significant. From (C) to (F), statistical analysis was performed using an unpaired two‐tailed Student's *t* test.

To uncover the underlying downstream effectors following ALKBH1 depletion and METTL3‐mediated m^6^A modification, RNA‐seq was performed in negative controls and ALKBH1‐depleted HCT116 cells. Analysis of Kyoto Encyclopedia of Genes and Genomes (KEGG) enrichment displayed that various pathways were changed in ALKBH1‐depleted cells (Fig. S11). Given that ALKBH1 affects METTL3 expression and METTL3‐mediated m^6^A modification, we compared these genes with the previous METTL3 knockdown m^6^A‐seq dataset and METTL3 knockdown RNA‐seq dataset to explore the potential downstream targets [27]. As shown, 4 overlapping genes were identified (ALKBH1 RNA‐seq, *P* < 0.5; |log2 fold change| > 1. METTL3 RNA‐seq, *P* < 0.5; |log2 fold change| > 0.2. METTL3 m^6^A‐seq, *P* < 0.5; log2 fold change < −0.5) (Fig. 6A,B). Consistent with the ALKBH1 RNA‐seq results, the mRNA levels of *SMAD7*, *CSRNP1*, *TRIM15*, and *RHOB* were dramatically increased in ALKBH1‐depleted HCT116 cells compared with normal controls (Fig. 6C). Moreover, MeRIP‐qPCR assays were performed to detect the specific m^6^A abundance and m^1^A abundance in these 4 transcripts. Knocking down ALKBH1 increased the m^1^A abundance in *METTL3* mRNA (Fig. 6D). Interestingly, ALKBH1 downregulation reduced the m^6^A abundance but not the m^1^A abundance in *SMAD7* mRNA (Fig. 6D,E). As expected, METTL3 depletion also inhibited *SMAD7* m^6^A abundance (Fig. 6F). However, the alterations in the m^1^A and m^6^A levels were not consistent in the other 3 genes (*CSRNP1*, *TRIM15*, and *RHOB*) (Fig. 6D–F). We further confirmed these findings about SMAD7 in RKO cells (Fig. S12A–E). Thus, SMAD7 was selected as a presumptive target of ALKBH1 for further investigation. Besides, we investigated the expression of *SMAD7* in CRC using GEPIA, which indicated that *SAMD7* expression was lower in tumor tissues compared to normal tissues in COAD, as well as READ (Fig. S13A). The analysis of the correlation between the OS in CRC patients and the expression of *SMAD7* in KM plotter showed that CRC patients with decreased *SMAD7* mRNA levels had worse overall survival (Fig. S13B).

Subsequently, we measured SMAD7 expression at the protein level. Knockdown of ALKBH1 enhanced SMAD7 expression in both HCT116 and RKO cells (Fig. 6G, Fig. S12F). Moreover, the SMAD7 level was significantly decreased in HCT116 and RKO cells overexpressing ALKBH1 but not in those overexpressing the catalytic mutant ALKBH1 (Fig. 6H, Fig. S12G). Depletion of METTL3 enhanced SMAD7 expression in HCT116 and RKO cells (Fig. 6I, Fig. S12H). Additionally, exogenic expression of ALKBH1‐wt but not the catalytically inactive ALKBH1 mutant reversed the increase in SMAD7 expression induced by ALKBH1 depletion (Fig. 6J, Fig. S12I). Moreover, ectopic expression of METTL3 reversed the increase in SMAD7 expression induced by METTL3 depletion (Fig. 6K, Fig. S12J). Further analysis showed that the upregulation of SMAD7 protein expression in ALKBH1‐depleted cells was reversed by ectopic expression of METTL3 (Fig. 6L, Fig. S12K). All these results suggest that SMAD7 is a downstream target of ALKBH1 and that ALKBH1 may regulate SMAD7 expression via METTL3‐mediated m^6^A modification.

### 3.7 ALKBH1 accelerates CRC metastasis by downregulating SMAD7 expression

**Fig. 7:**
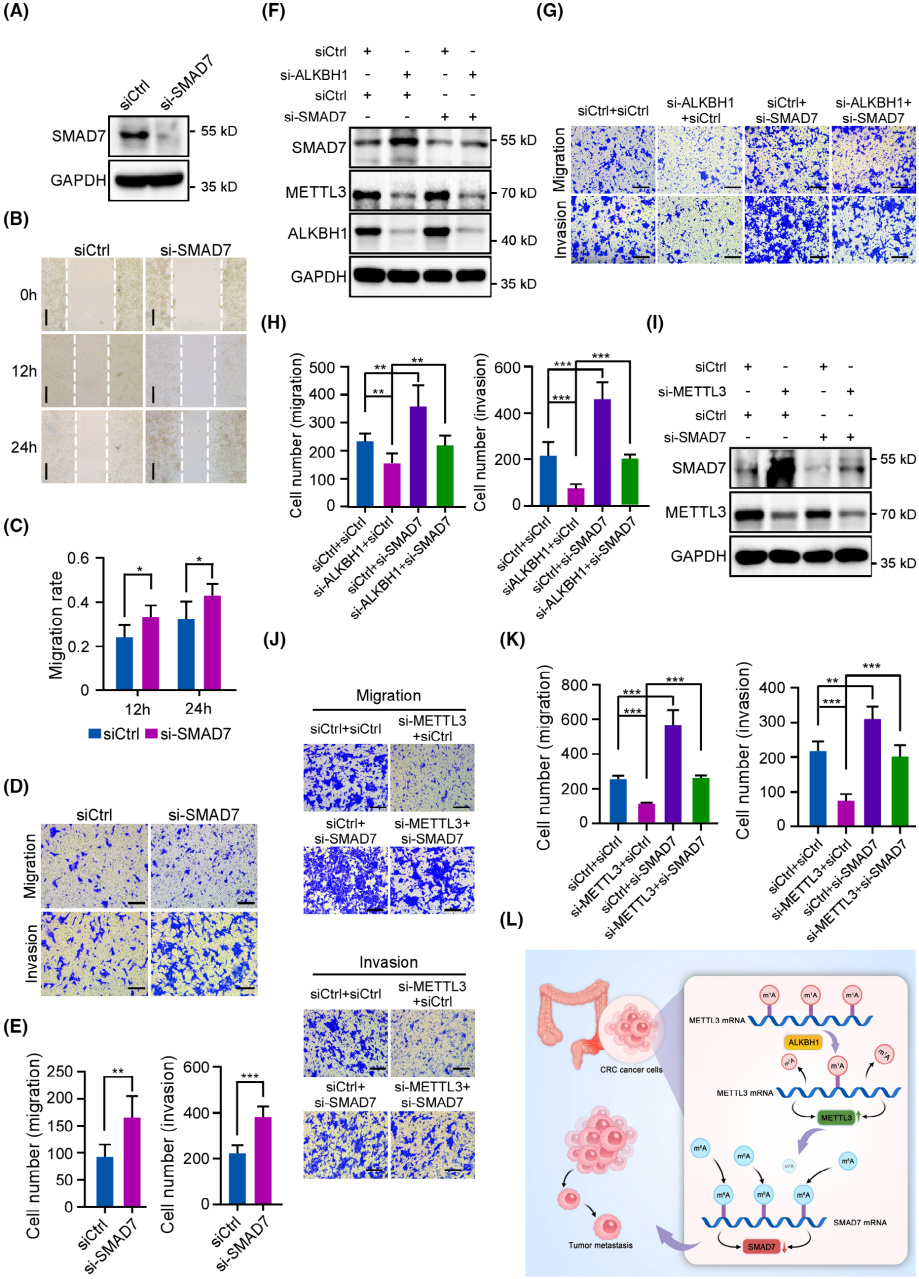
ALKBH1 boosts colorectal cancer (CRC) metastasis by downregulating SMAD7 expression. HCT116 cells transfected with the indicated siRNAs and vectors were subjected to the following analyses: (A) HCT116 cells were transfected with control or SMAD7 siRNAs for 48 h and then subjected to western blot analysis with anti‐SMAD7 antibody. GAPDH, a loading control. Three independent experiments were performed. (B, C) The wound healing assays displayed cell migration at the different time points. Dashed lines indicate the wound edges. Scale bar, 200 μm. The distance between the two edge lines was measured by imagej software. Comparisons were analyzed by an unpaired two‐tailed Student's *t* test. Three independent experiments were performed. (D, E) Transwell migration and invasion assays revealed the cell migration and invasion abilities of control and SMAD7‐depleted cells. Scale bar, 200 μm. Cells that moved to the undersides of the filters were counted. Comparisons were analyzed by an unpaired two‐tailed Student's *t* test. Experiments were performed in triplicate. (F) Western blotting analysis of the expression of SMAD7, METTL3 and ALKBH1. GAPDH was served as a loading control. Experiments were performed in triplicate. (G, H) Transwell migration and invasion assays revealed the cell migration and invasion abilities. Scale bar, 200 μm. Cells that migrated to the undersides of the filters were counted. Comparisons were analyzed by one‐way ANOVA. Experiments were performed in triplicate. (I) Western blotting analysis of the expression of SMAD7 and METTL3. GAPDH was served as a loading control. Experiments were performed in triplicate. (J, K) Transwell migration and invasion assays revealed the cell migration and invasion abilities. Scale bar, 200 μm. Cells that migrated to the undersides of the filters were counted. Comparisons were analyzed by one‐way ANOVA. Experiments were performed in triplicate. Quantitative data from three independent experiments are shown as the mean ± SD. *P < 0.05; **P < 0.01; ***P < 0.001. (L) Working model of ALKBH1 promoting CRC metastasis by destabilizing SMAD7 through METTL3.

To further characterize the function of SMAD7 in CRC cells, we designed a siRNA targeting *SMAD7* mRNA and confirmed the knockdown efficiency by western blot analysis (Fig. 7A). The results showed that knockdown of SMAD7 dramatically promoted the migration and invasion of HCT116 and RKO cells (Fig. 7B–E, Fig. S14A–E). Furthermore, depletion of SMAD7 rescued the reduced migration and invasion abilities of ALKBH1 knockdown CRC cells (Fig. 7F–H, Fig. S14F–H). More importantly, as expected, the expression of SMAD7 was knocked down in METTL3‐depleted CRC cells using a *SMAD7*‐specific siRNA (Fig. 7I, Fig. S14I), which markedly promoted the suppression of migration and invasion resulting from depletion of METTL3 (Fig. 7J,K, Fig. S14J,K). Finally, we performed IHC assays to further analyze the correlation between the expressions of ALKBH1, METTL3, and SMAD7 in 20 CRC tissue samples. The results showed a positive correlation between ALKBH1 and METTL3 expression at the protein level (Fig. S15B, representative images are shown in Fig. S15A). Besides, a significant negative correlation between ALKBH1 and SMAD7 expression in protein levels was observed (Fig. S15C, representative images are shown in Fig. S15A). We further confirmed that METTL3 levels were negatively correlated to SMAD7 expression in CRC tissues (Fig. S15D, representative images are shown in Fig. S15A). Thus, our data indicate that ALKBH1 promotes CRC metastasis through the degradation of SMAD7.

## 4 Discussion

ALKBH1 is a 2‐oxoglutarate and Fe^2+^‐dependent dioxygenase. Accumulating studies indicate that ALKBH1 is related to a range of cancers, although its role in different cancers is inconsistent. For example, low ALKBH1 expression is related to the poor prognosis of patients with pancreatic cancer [28]. While in most tumors, ALKBH1 works as an oncogene. For instance, in glioblastoma, the DNA demethylase ALKBH1 regulates N^6^‐mA levels dynamically, and its depletion leads to transcriptional silencing of oncogenic pathways by decreasing chromatin accessibility [29]. In gastric cancer, ALKBH1 is overexpressed, and its overexpression markedly enhances viability and migration [30]. Here, we show for the first time that ALKBH1 is upregulated in CRC and that elevated ALKBH1 expression correlates with poor prognosis in patients with CRC. ALKBH1 depletion impedes the migration and invasion of CRC cells. Additionally, overexpression of ALKBH1 boosts CRC cell migration and invasion, while catalytic mutant ALKBH1 has no such effects. Furthermore, ectopic expression of wild‐type ALKBH1 but not mutant ALKBH1 reverses the defects in cell migration and invasion induced by ALKBH1 depletion. To the best of our knowledge, these findings indicate that ALKBH1 plays an important role in facilitating CRC metastasis.

Mounting evidence supports a critical role of ALKBH1 in a list of posttranscriptional modifications, including m^6^A, m^1^A, m^3^C, m^5^C, and N^6^‐mA [11, 25, 29, 31, 32]. Previous studies have shown that ALKBH1 may participate in the occurrence and development of pancreatic cancer through the mTOR and ErbB signaling pathways as an m^1^A regulator [28]. In addition, ALKBH1 is an m^1^A demethylase that leads to destabilization of tRNA and enhances tRNA cleavage [33]. Additionally, ALKBH1 may remove N^6^‐mA modifications on transcription factor 4 (ATF4), HIF‐1α, and GYS1, which activate the ATF4 and HIF‐1 pathways, respectively [34, 35]. In this report, we find that depleted ALKBH1 reduces the expression of METTL3. Further data also show that ALKBH1 downregulation leads to an increase of SMAD7. Exogenous expression of METTL3 reverses the upregulation of SMAD7 in ALKBH1‐depleted cells. Importantly, depletion of SMAD7 is able to reverse the decreases in cell migration and invasion induced by ALKBH1 depletion. Taken together, these data indicate that ALKBH1 regulates SMAD7 expression through METTL3.

METTL3 is the most abundant m^6^A methyltransferase. Extensive evidence obtained in recent years has revealed that METTL3 plays key roles in various types of cancer, either dependent or independent of its m^6^A RNA methyltransferase activity. Especially, it' is reported that METTL3 was frequently upregulated in CRC tissues and METTL3 is highly expressed in metastatic CRC and associated with poor prognosis [27, 36, 37]. Previous studies have shown that cigarette smoke condensate induces hypomethylation of the METTL3 promoter and, subsequently, recruitment of the transcription factor *NFIC* to induce METTL3 overexpression in pancreatic cancer [38]. It has been demonstrated that P300 regulates histone H3 acetylation at lysine 27 (H3K27ac) and promotes METTL3 transcription in gastric cancer [39]. Additionally, several microRNAs, such as miR‐186, have been proposed to suppress METTL3 expression by targeting *METTL3* mRNA [40]. Here, we find that the METTL3 protein level is substantially decreased in ALKBH1‐depleted HCT116 and RKO cells. Overexpression of ALKBH1 promotes METTL3 expression, but this promotion is not observed in mutant ALKBH1‐overexpressing cells. Moreover, upregulated ALKBH1 not only enhances the protein expression of METTL3 but also increases its m^1^A abundance. Together, these data suggest that ALKBH1 may regulate METTL3 expression by mediating METTL3 m^1^A modification.

It has been shown that ALKBH1 can catalyze tRNA m^1^A demethylation, which affects translation initiation, elongation, and protein synthesis [11]. Interestingly, reports have shown that m^1^A in mitochondrial mRNA interferes with translation [41]. Moreover, mitochondrial protein synthesis is substantially reduced in ALKBH1 KO cells in comparison to WT cells [42]. However, the effect of m^1^A mRNA modification on translation is still unclear. In our study, the data reveal that ALKBH1 depletion only reduces the protein level of METTL3 and not the *METTL3* mRNA level. Moreover, ALKBH1 did not affect the stability of METTL3 protein. Thus, we suspect that ALKBH1 may promote the translation of METTL3 by mediating the m^1^A level in *METTL3* mRNA. However, the detailed mechanism is unclear. We will explore this issue more thoroughly in the future using polysome fractionation and isotope labeling technology as well as glucose deprivation assays. GUUCRA (“R” = G or A) is a common motif that undergoes m^1^A modification in mRNA [41]. In this report, we find that knockdown of ALKBH1 increases the m^1^A abundance in *METTL3* mRNA, although the specific m^1^A modification site in *METTL3* mRNA is not clear. In addition, m^6^A readers have been reported to recognize m^6^A sites, resulting in different mRNA fates [43]. Previous studies have shown that the readers YTHDC1, YTHDF1‐3, IGF2BP1‐3, and HuR are involved in the regulation process dependent on METTL3‐mediated m^6^A modification [44]. Whether a reader participates in the inhibitory regulation of SMAD7 by METTL3 should be investigated in the future.

Accumulating studies have shown that SMAD7 is an intracellular protein, which has been traditionally considered as a negative regulator of transforming growth factor (TGF)‐β1 [45, 46, 47]. TGF‐β1 seems to play both pro‐tumorigenic and anti‐tumorigenic roles in CRC depending on the tumor stage [45, 46]. Thus, a dual role of SMAD7 has been described in various types of cancer with pro‐tumorigenic or anti‐tumorigenic effects depending on the cancer site and biology [46, 48]. Previous studies have shown that SMAD7 was a tumor suppressor in a variety of cancers. For instance, depletion of SMAD7 in lung cancer cells markedly increased transwell migration and invasion [49]. Another *in vivo* study showed that SMAD7 overexpression inhibited the lung metastasis of melanoma cells [50]. As to CRC, recent studies indicated that depletion of SMAD7 induced a TGF‐β1‐dependent epithelial–mesenchymal transition, thus promoting CRC metastatic process [51, 52]. In this study, the anti‐invasive role of SMAD7 could explain how ALKBH1 promotes the invasion of CRC cells. However, whether TGF‐β1 was involved in this process needs further experiments.

## 5 Conclusion

In summary, we identified the important role of ALKBH1‐catalyzed m^1^A modification in CRC metastasis. ALKBH1 expression is increased in CRC and associated with poor survival. Depletion of ALKBH1 leads to a decrease in METTL3 expression, probably by methylating the m^1^A site in *METTL3* mRNA, further causing m^6^A demethylation in *SMAD7* mRNA. In addition, downregulation of SMAD7 manifests an aggressive phenotype, in which the defects in CRC cell migration and invasion caused by ALKBH1 depletion or METTL3 depletion are reversed. Considering these results collectively, we propose that ALKBH1 may promote CRC metastasis by destabilizing SMAD7 through METTL3 (Fig. 7L).

## Conflict of interest

The authors declare no conflict of interest.

## Author contributions

WC, HW, and SM carried out the experiments and analyzed the data. WC and MX designed experiments. WC wrote the manuscript. MX edited the manuscript. MX and ZX supervised the project. ZX and LS helped with clinical sample collection. All authors read and approved the manuscript.

### Peer review

The peer review history for this article is available at https://publons.com/publon/10.1002/1878‐0261.13366.

## Supporting information


**Fig. S1.** Survival curves of overall survival (OS) based on *TRMT6*, *TRMT61A*, *TRMT61B*, or *ALKBH3* expression using the online bioinformatics tool GenomicScape.
**Fig. S2.** Analyses of m^1^A methyltransferases and demethylases mRNA levels of colon adenocarcinoma (COAD) and rectum adenocarcinoma (READ) tissues using the online bioinformatics tool Gene Expression Profiling Interactive Analysis (GEPIA).
**Fig. S3.** Representative staining images of ALKBH1 for each score were shown.
**Fig. S4.** ALKBH1 expression level in colorectal cancer (CRC) cell lines and the migratory ability of CRC cells are shown.
**Fig. S5.** Depletion of ALKBH1 has no effect on colorectal cancer (CRC) cell viability in HCT116 cells.
**Fig. S6.** Depletion of ALKBH1 has no effect on colorectal cancer (CRC) cell viability in RKO cells.
**Fig. S7.** ALKBH1 accelerates the migration and invasion of RKO cells through its m^1^A demethylation activity.
**Fig. S8.** ALKBH1 affects METTL3 protein expression and METTL3‐mediated m^6^A modification.
**Fig. S9.** Knockdown of ALKBH1 had no significant effect on the *METTL3* mRNA level.
**Fig. S10.** ALKBH1 did not affect the METTL3 protein stability.
**Fig. S11.** Kyoto Encyclopedia of Genes and Genomes (KEGG) enrichment analysis of the differential genes in ALKBH1‐depleted cells compared with negative control cells. Top 20 terms were displayed.
**Fig. S12.** ALKBH1‐mediated m^1^A demethylation of *METTL3* mRNA inhibits SMAD7 expression by METTL3‐mediated m^6^A modification in RKO cells.
**Fig. S13.** SMAD7 is downregulated in colorectal cancer (CRC) tissues and related to the poor prognosis in patients.
**Fig. S14.** ALKBH1 boosts the invasiveness of RKO cells by downregulating SMAD7 expression.
**Fig. S15.** The clinical correlation between ALKBH1, METTL3 and SMAD7 expression in colorectal cancer (CRC) tissue samples.

## Data Availability

All the data that support the findings of this study are available from the corresponding author [xuemeng@zju.edu.cn] upon reasonable request.
