# Supplementary material for: ALKBH1‐mediated m1A demethylation of METTL3 mRNA promotes the metastasis of colorectal cancer by downregulating SMAD7 expression
Source: Mol Oncol. 2022 Dec 30;17(2):344–64. doi: 10.1002/1878-0261.13366 (PMC9892827; doi:10.1002/1878-0261.13366)
Supplement: Supplementary file 1 — Fig. S1. Survival curves of overall survival (OS) based on TRMT6, TRMT61A, TRMT61B, or ALKBH3 expression using the online bioinformatics tool GenomicScape. Fig. S2. Analyses of m1A methyltransferases and demethylases mRNA levels of colon adenocarcinoma (COAD) and rectum adenocarcinoma (READ) tissues using the online bioinformatics tool Gene Expression Profiling Interactive Analysis (GEPIA). Fig. S3. Representative staining images of ALKBH1 for each score were shown. Fig. S4. ALKBH1 expression level in colorectal cancer (CRC) cell lines and the migratory ability of CRC cells are shown. Fig. S5. Depletion of ALKBH1 has no effect on colorectal cancer (CRC) cell viability in HCT116 cells. Fig. S6. Depletion of ALKBH1 has no effect on colorectal cancer (CRC) cell viability in RKO cells. Fig. S7. ALKBH1 accelerates the migration and invasion of RKO cells through its m1A demethylation activity. Fig. S8. ALKBH1 affects METTL3 protein expression and METTL3‐mediated m6A modification. Fig. S9. Knockdown of ALKBH1 had no significant effect on the METTL3 mRNA level. Fig. S10. ALKBH1 did not affect the METTL3 protein stability. Fig. S11. Kyoto Encyclopedia of Genes and Genomes (KEGG) enrichment analysis of the differential genes in ALKBH1‐depleted cells compared with negative control cells. Top 20 terms were displayed. Fig. S12. ALKBH1‐mediated m1A demethylation of METTL3 mRNA inhibits SMAD7 expression by METTL3‐mediated m6A modification in RKO cells. Fig. S13. SMAD7 is downregulated in colorectal cancer (CRC) tissues and related to the poor prognosis in patients. Fig. S14. ALKBH1 boosts the invasiveness of RKO cells by downregulating SMAD7 expression. Fig. S15. The clinical correlation between ALKBH1, METTL3 and SMAD7 expression in colorectal cancer (CRC) tissue samples. [file MOL2-17-344-s001.docx]

**Supplementary Figures**

**Fig. S1**

**
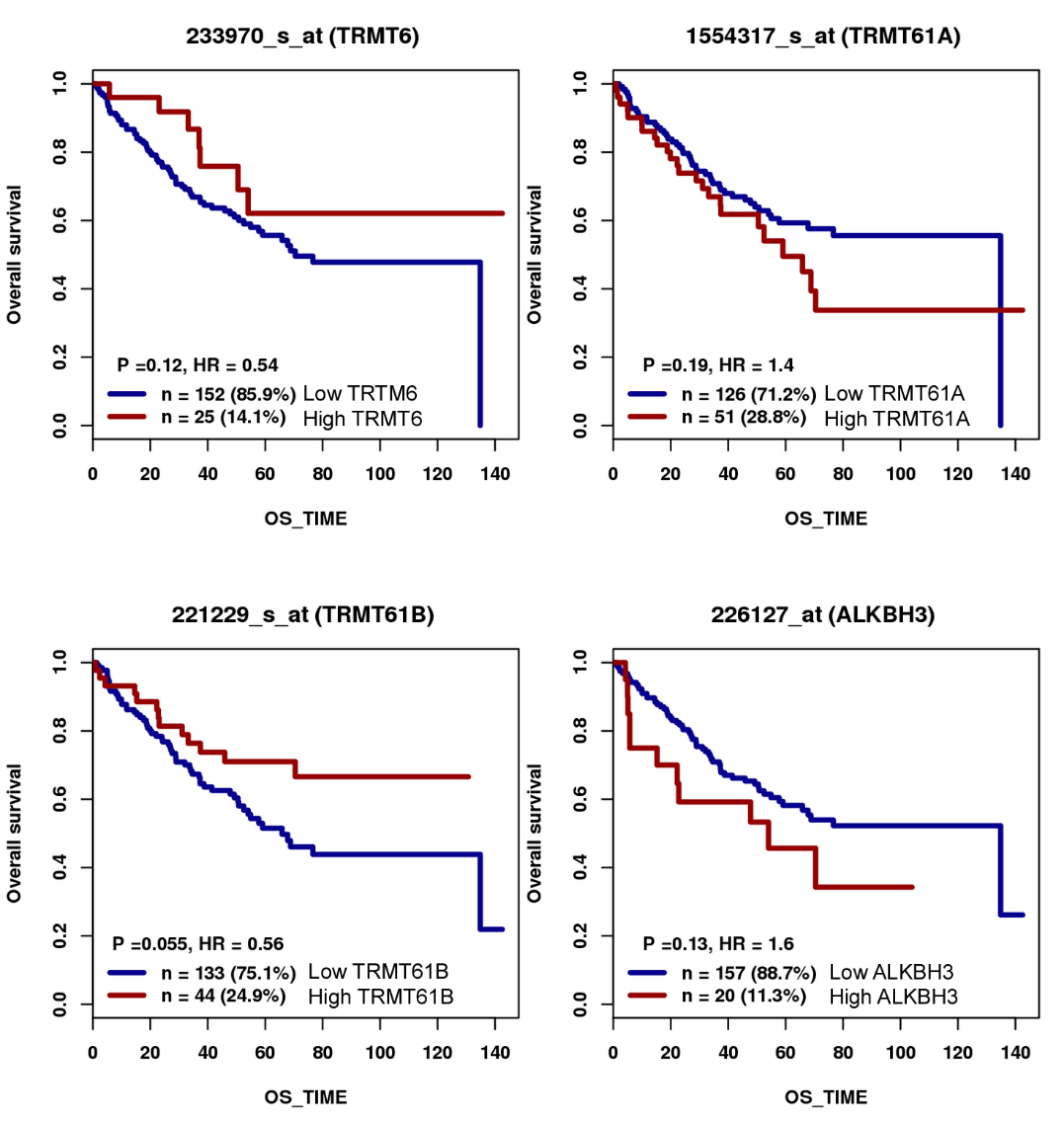
**

**Figure. S1.** Survival curves of overall survival (OS) based on *TRMT6*, *TRMT61A*, *TRMT61B* or *ALKBH3* expression using the online bioinformatics tool GenomicScape.

**Fig. S2**


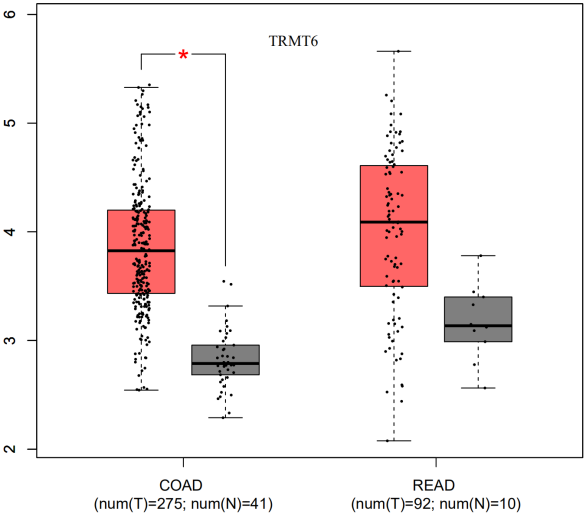

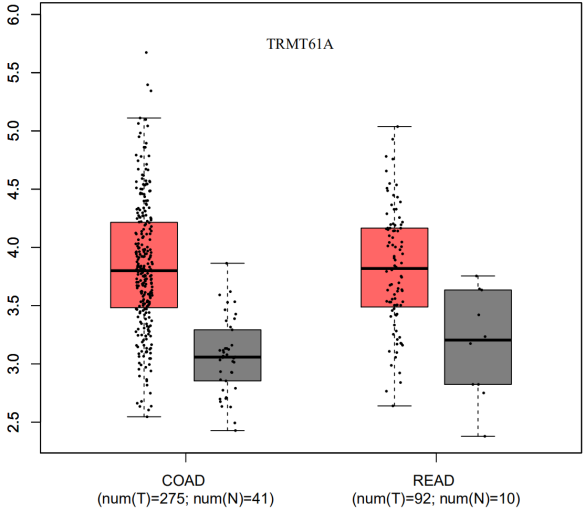


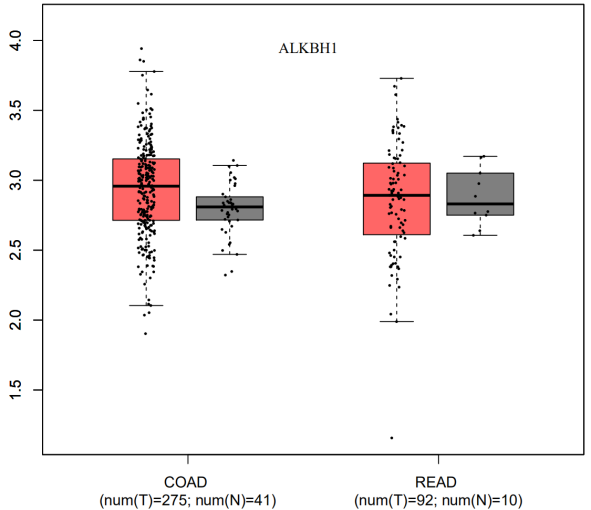

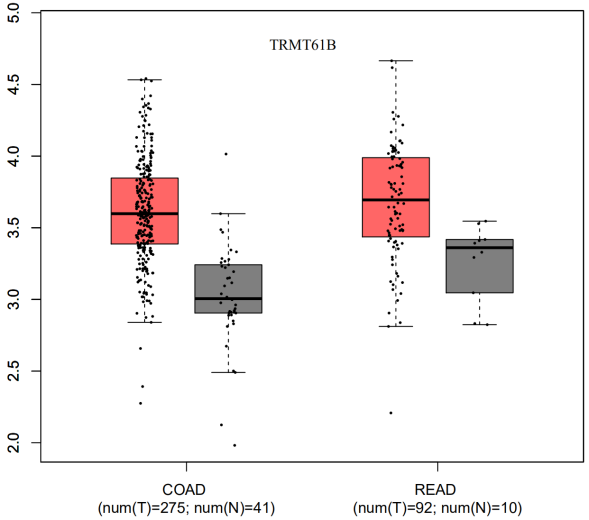


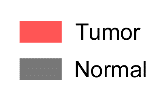

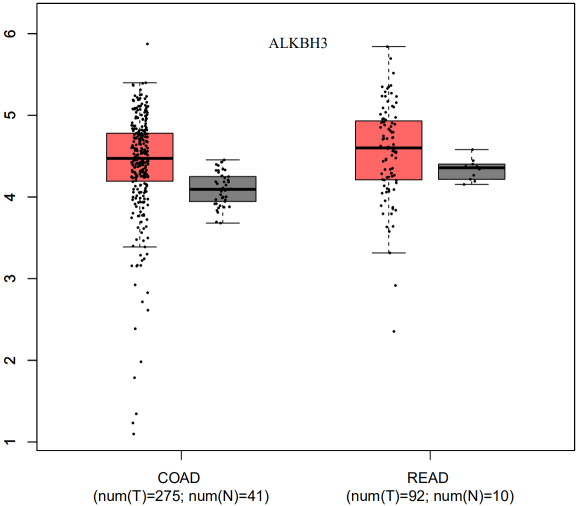


**Figure. S2.** Analyses of m^1^A methyltransferases and demethylases mRNA levels of colon adenocarcinoma (COAD) and rectum adenocarcinoma (READ) tissues using the online bioinformatics tool Gene Expression Profiling Interactive Analysis (GEPIA).

**Fig. S3**


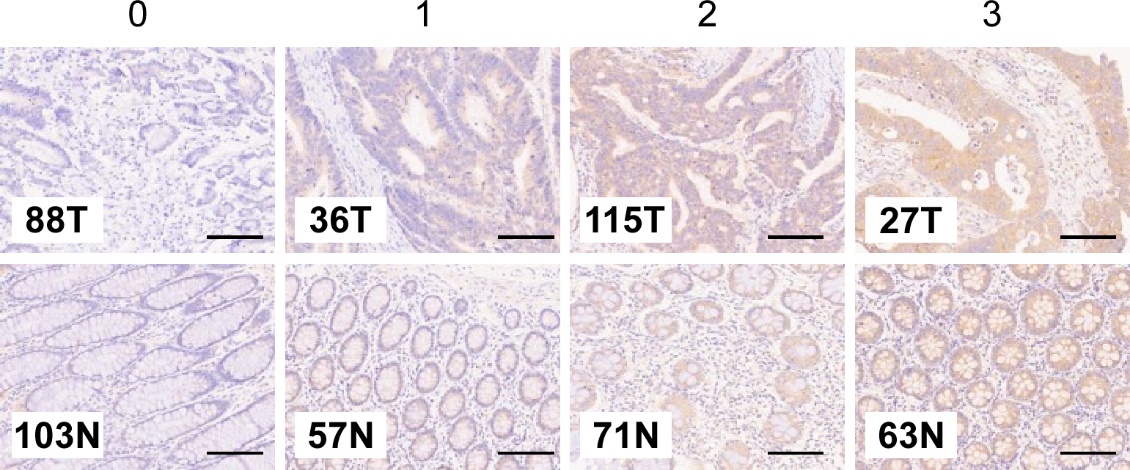


**Figure. S3.** Representative staining images of ALKBH1 for each score were shown. Scale bar, 100 µm.

**Fig. S4**


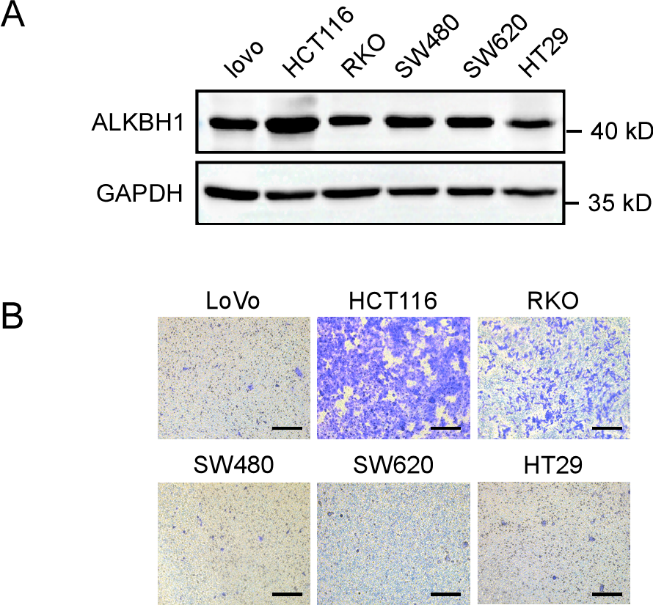


**Figure. S4. ALKBH1 expression level in colorectal cancer (CRC) cell lines and the migratory ability of CRC cells are shown.** (A) Western blot analysis of the expression of ALKBH1 in wild CRC cells. GAPDH was used as a loading control. (B) Transwell migration assays revealed the cell migration ability in wild CRC cells. Scale bar, 200 µm.

**Fig. S5**


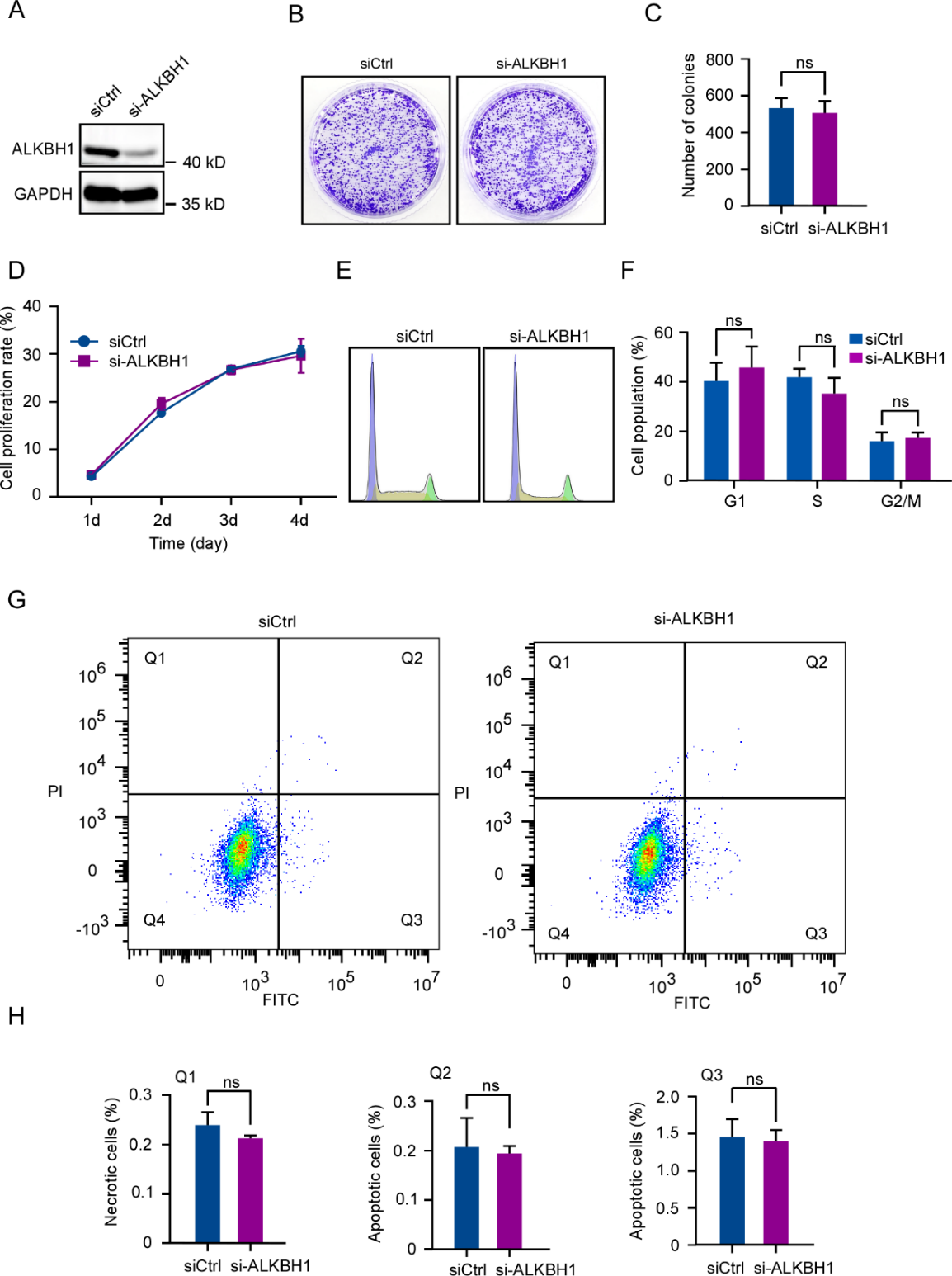


**Figure. S5. Depletion of ALKBH1 has no effect on colorectal cancer (CRC) cell viability in HCT116 cells.** (A) HCT116 cells were transfected with control or ALKBH1 siRNAs for 48 h and then subjected to western blot analysis with anti-ALKBH1 antibody. GAPDH was used as a loading control. (B-C) Colony‐forming growth assays revealed the cell proliferation ability of control and ALKBH1-depleted cells. Colonies were captured and counted. Statistical analysis was performed using an unpaired Two-tailed Student’s t test. (D) CCK8 assays were performed to detect the cell proliferation ability of control and ALKBH1-depleted cells. (E, F) Flow cytometry analysis of the cell cycle distribution in control and ALKBH1-depleted cells. Statistical analysis was performed using an unpaired Two-tailed Student’s t test. (G, H) Flow cytometry analysis of the cell apoptosis was displayed in control and ALKBH1-depleted cells. Statistical analysis was performed using an unpaired Two-tailed Student’s t test. ns not significant.

**Fig. S6**


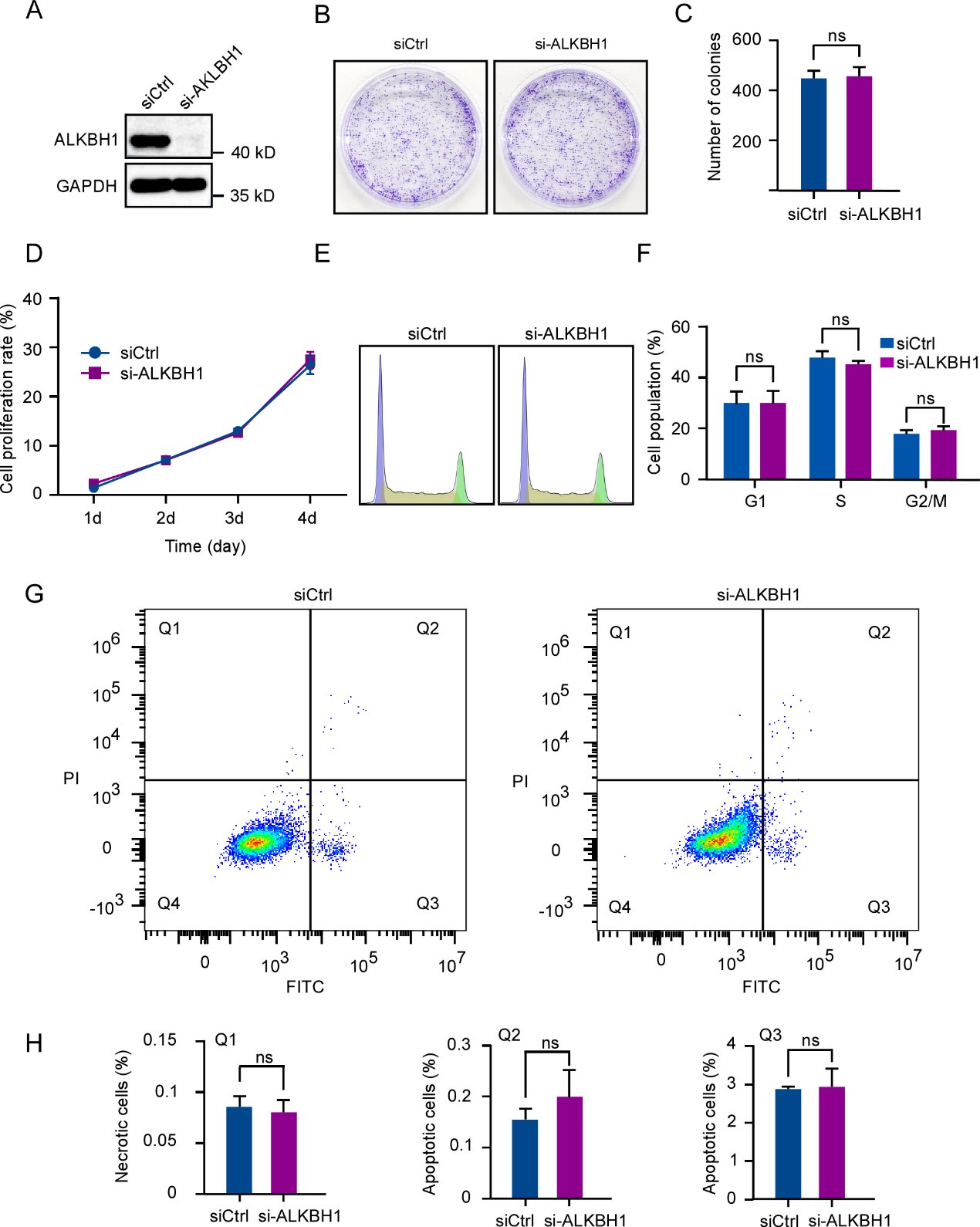


**Figure. S6. Depletion of ALKBH1 has no effect on colorectal cancer (CRC) cell viability in RKO cells.** (A) RKO cells were transfected with control or ALKBH1 siRNAs for 48 h and then subjected to western blot analysis with anti-ALKBH1 antibody. GAPDH was used as a loading control. (B-C) Colony‐forming growth assays revealed the cell proliferation ability of control and ALKBH1-depleted cells. Colonies were captured and counted. Statistical analysis was performed using an unpaired Two-tailed Student’s t test. (D) CCK8 assays were performed to detect the cell proliferation ability of control and ALKBH1-depleted cells. (E, F) Flow cytometry analysis of the cell cycle distribution in control and ALKBH1-depleted cells. Statistical analysis was performed using an unpaired Two-tailed Student’s t test. (G, H) Flow cytometry analysis of the cell apoptosis was displayed in control and ALKBH1-depleted cells. Statistical analysis was performed using an unpaired Two-tailed Student’s t test. ns not significant.

**Fig. S7**


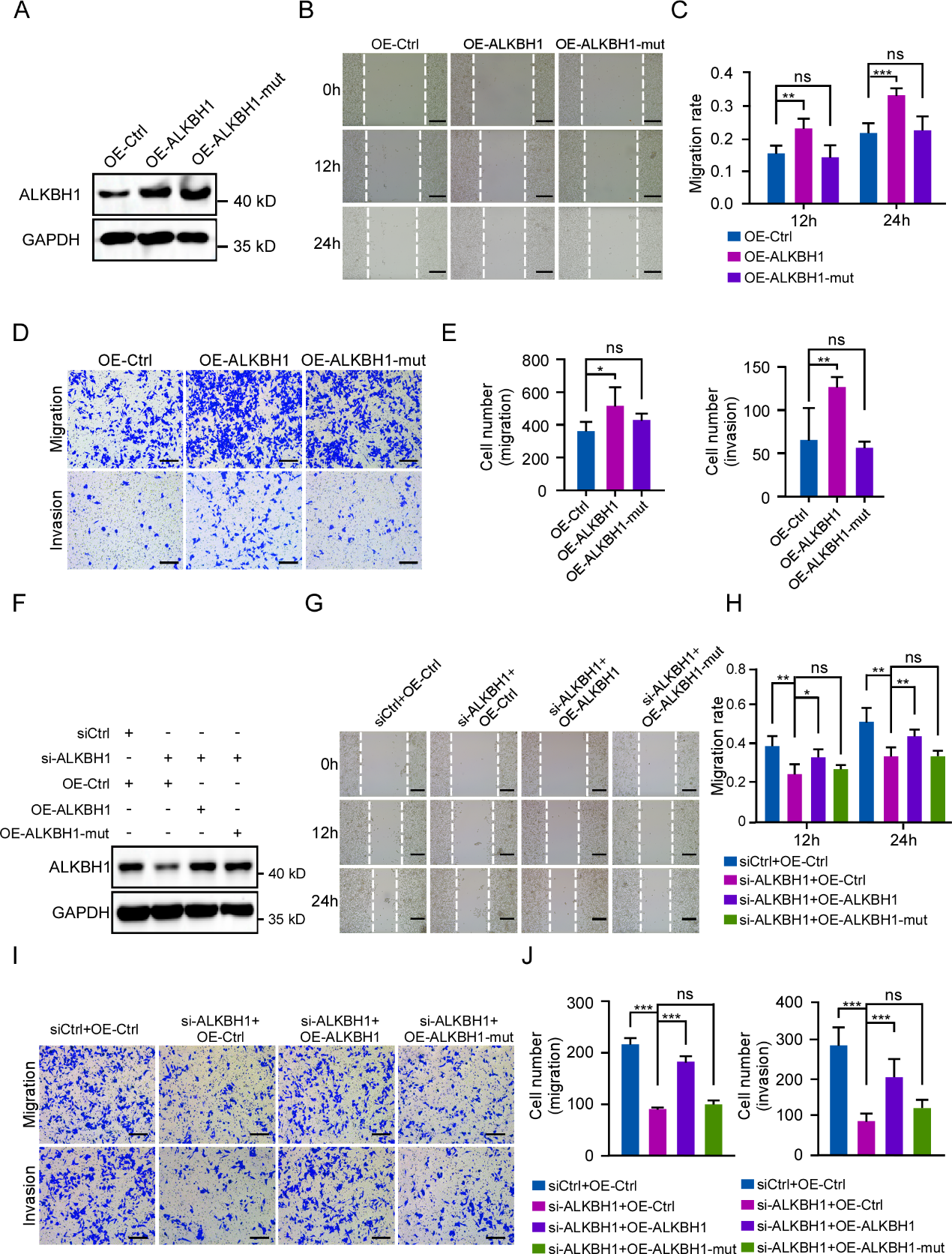


**Figure. S7. ALKBH1 accelerates the migration and invasion of RKO cells through its m^1^A demethylation activity.** (A) RKO cells transfected with control, ALKBH1 and mutant ALKBH1 plasmids for 48 h were subjected to western blot analysis with anti-ALKBH1 antibody. GAPDH, a loading control. (B, C) The wound healing assays revealed cell migration at the different time points. Dashed lines indicate the wound edges. Scale bar, 200 µm. The distance between the two edge lines was measured by ImageJ software. Comparisons were analyzed by One-way ANOVA. (D, E) Transwell migration assays and transwell invasion assays revealed the cell migration and invasion abilities. Scale bar, 200 µm. Cells that migrated to the undersides of the filters were counted. Comparisons were analyzed by One-way ANOVA. (F) RKO cells transfected with the indicated siRNAs and plasmids were subjected to western blot analysis with anti-ALKBH1 antibody. GAPDH was used as a loading control. (G, H) Wound healing assays detected cell migration at the different time points. The distance between the two edge lines was measured using ImageJ software. Scale bar, 200 µm. Comparisons were analyzed by One-way ANOVA. (I, J) Transwell migration and invasion assays were performed to detect cell migration and invasion. Scale bar, 200 µm. Cells that migrated to the undersides of the filters were counted. Comparisons were analyzed by One-way ANOVA. Quantitative data from three independent experiments are shown as the mean ± SD. **P* < 0.05; ***P* < 0.01; ****P* < 0.001; ns not significant.

**Fig. S8**


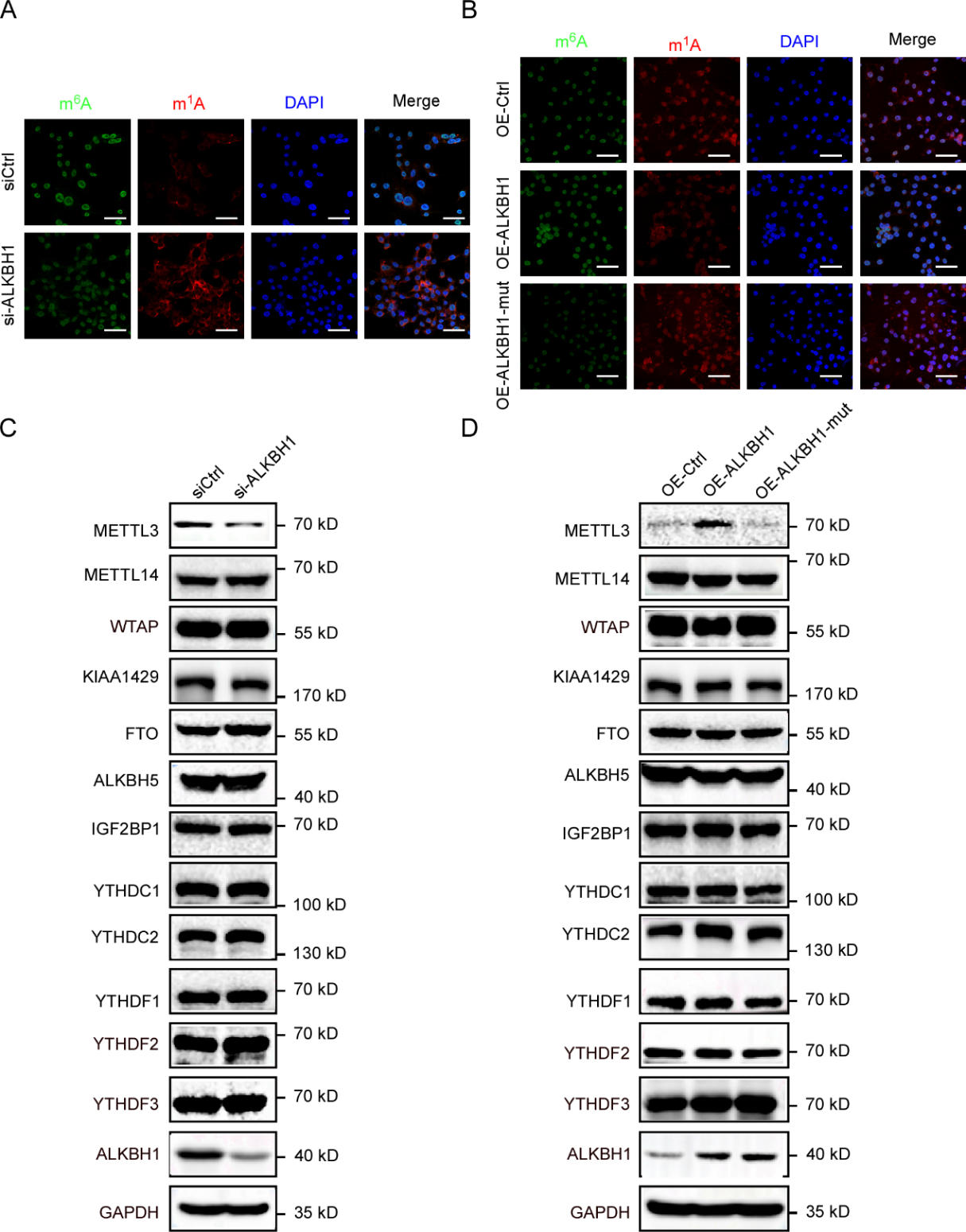


**Figure. S8. ALKBH1 affects METTL3 protein expression and METTL3-mediated m^6^A modification in RKO cells.** (A) RKO cells transfected with the indicated siRNAs were subjected to immunofluorescence. Cells were fixed and stained with anti-m^6^A (green) and anti-m^1^A (red). DNA was visualized with DAPI (blue). Scale bar, 30 µm. (B) RKO cells transfected with the indicated plasmids were subjected to immunofluorescence. Cells were fixed and stained with anti-m^6^A (green) and anti-m^1^A (red). DNA was visualized with DAPI (blue). Scale bar, 30 µm. (C, D) RKO cells transfected with the indicated siRNAs or vectors were subjected to western blot analysis with the indicated antibodies. GAPDH was used as a loading control. Experiments were performed in triplicate.

**Fig. S9**


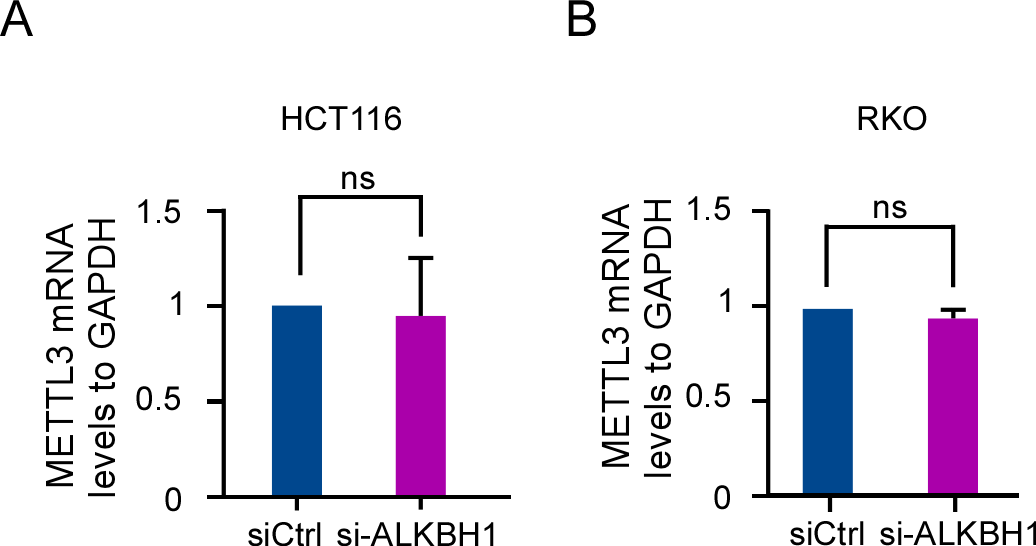


**Figure. S9.** **Knockdown of ALKBH1 had no significant effect on the *METTL3* mRNA level.** (A) Quantitative RT-PCR analysis of *METTL3* mRNA in control and ALKBH-depleted HCT116 cells. GAPDH was used as an internal control. (B) Quantitative RT-PCR analysis of *METTL3* mRNA in control and ALKBH-depleted RKO cells. GAPDH was used as an internal control. Quantitative data from three independent experiments are shown as the mean ± SD. ns not significant. The two-tailed Student’s t-test was used to perform comparison between two groups.

**Fig. S10**


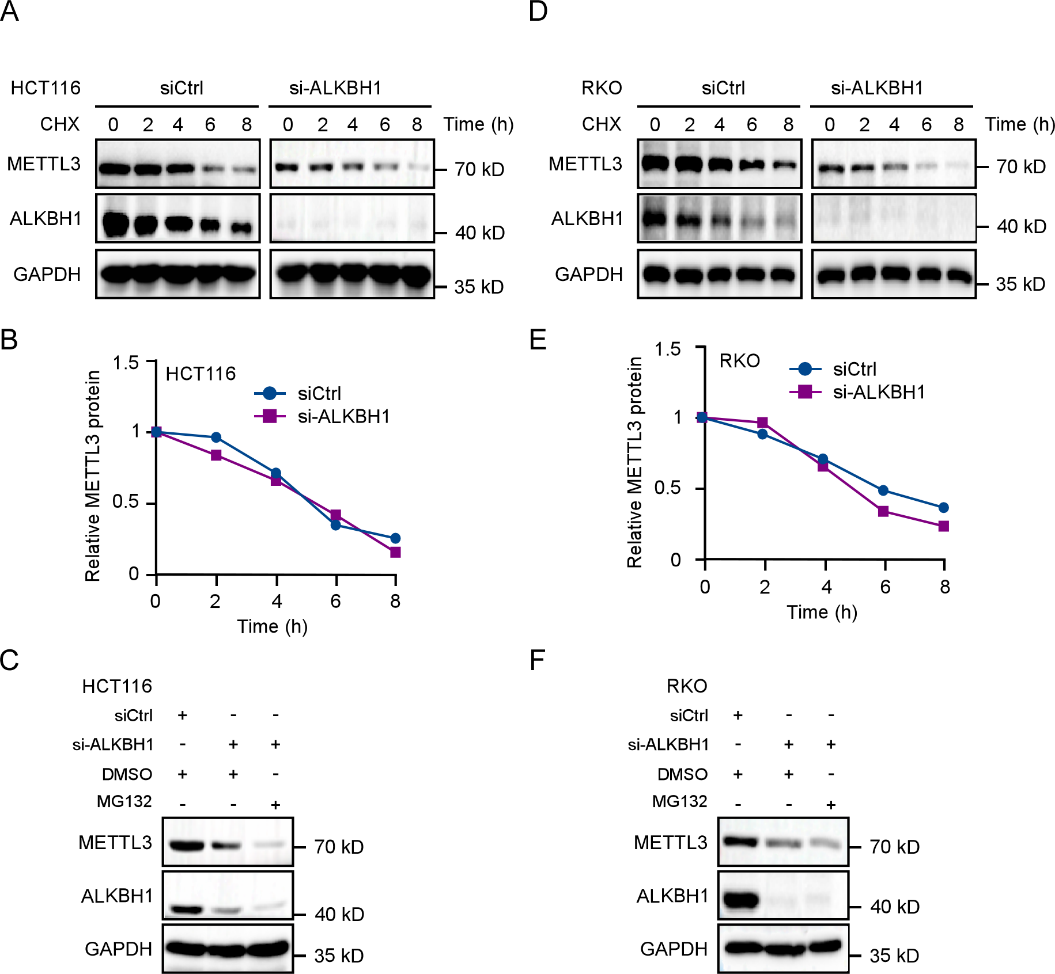


**Figure. S10.** **ALKBH1 did not affect the METTL3 protein stability.** (A) Control and ALKBH1-depleted HCT116 cells treated with 50 μg/ml Cycloheximide (CHX) were harvested at different time points. The cell lysates were subjected to western blot analysis using anti-METTL3 and anti-ALKBH1 antibodies. GAPDH, a loading control. (B) ImageJ software was used to quantify protein levels. The relative amounts of METTL3 were calculated after normalization (METTL3/GAPDH). (C) Control and ALKBH1-depleted HCT116 cells were treated with MG132 or dimethyl sulfoxide (DMSO) for 4 h. Lysates of the cells were applied for western blot analysis with anti-METTL3 and anti-ALKBH1 antibodies. GAPDH, a loading control. (D) Control and ALKBH1-depleted RKO cells treated with 50 μg/ml CHX were harvested at different time points. The cell lysates were subjected to western blot analysis using anti-METTL3 and anti-ALKBH1 antibodies. GAPDH, a loading control. (E) ImageJ software was used to quantify protein levels. The relative amounts of METTL3 were calculated after normalization (METTL3/GAPDH). (F) Control and ALKBH1-depleted RKO cells were treated with MG132 or DMSO for 4 h. Lysates of the cells were applied for western blot analysis with anti-METTL3 and anti-ALKBH1 antibodies. GAPDH, a loading control.

**Fig. S11**


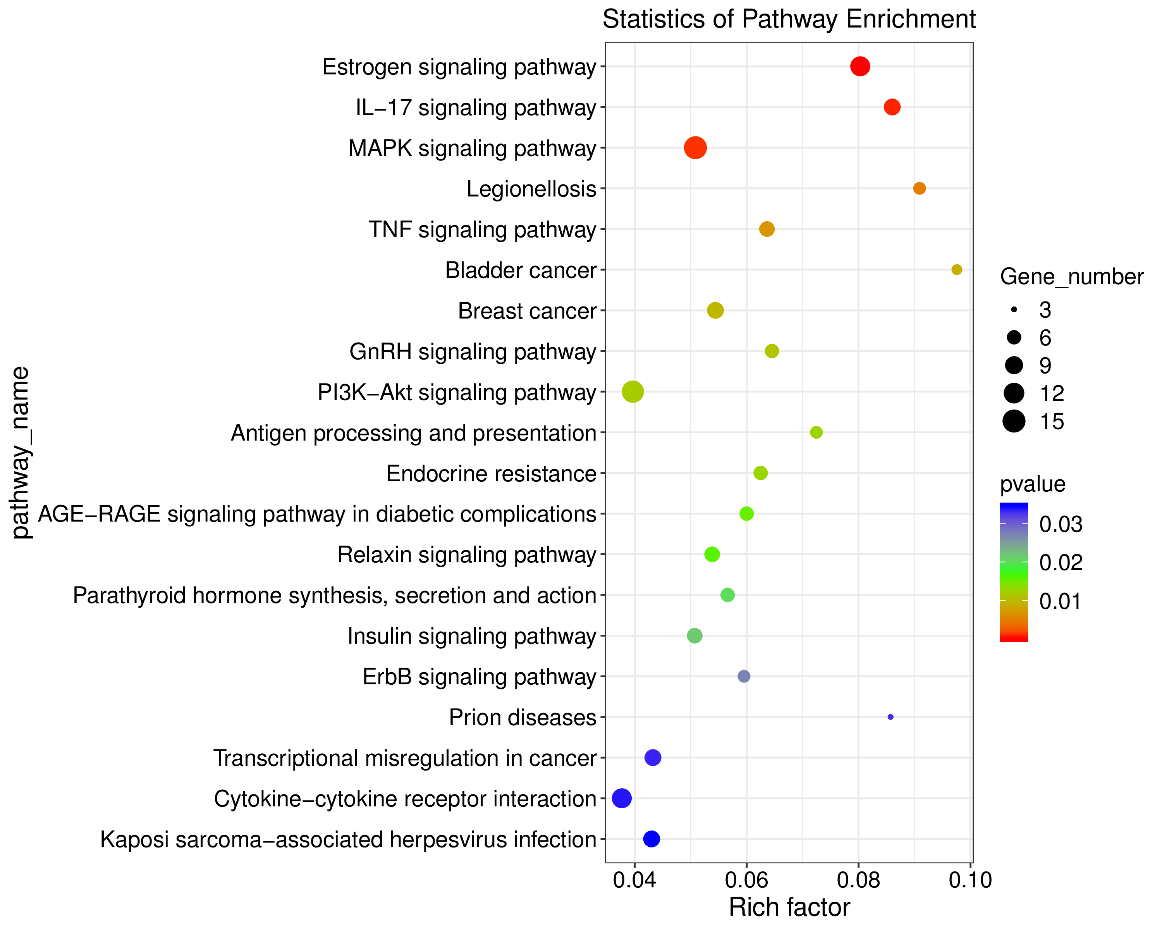


**Figure. S11.** Kyoto Encyclopedia of Genes and Genomes (KEGG) enrichment analysis of the differential genes in ALKBH1-depleted cells compared with negative control cells. Top 20 terms were displayed.

**Fig. S12**


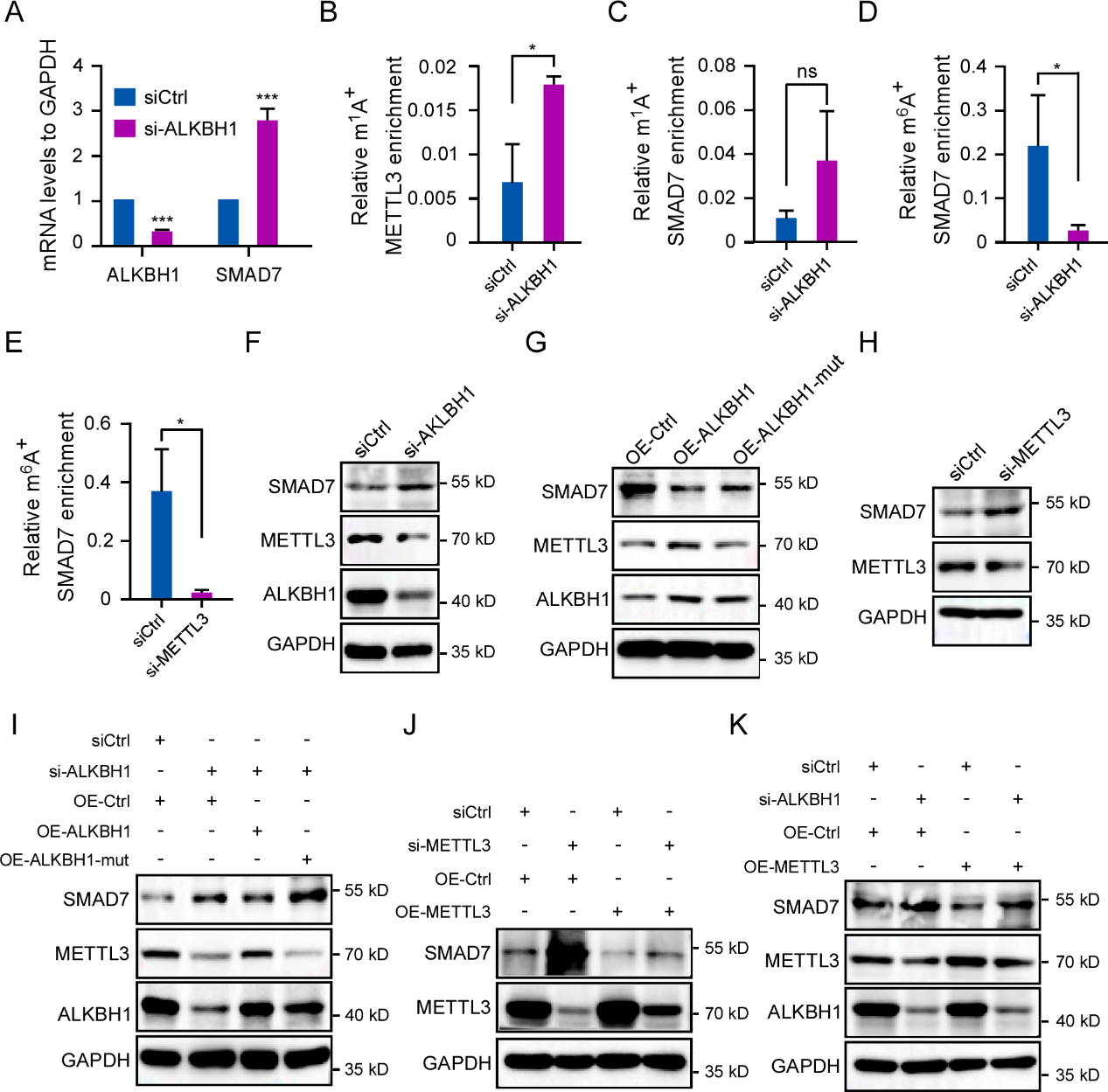


**Figure. S12.** **ALKBH1-mediated m^1^A demethylation of *METTL3* mRNA inhibits SMAD7 expression by METTL3-mediated m^6^A modification in RKO cells.** (A) Quantitative RT-PCR analysis of *ALKBH1* and *SMAD7* mRNA in control and ALKBH-depleted RKO cells. *GAPDH* was used as an internal control. Statistical analysis was performed using an unpaired Two-tailed Student’s t test. (B) Methylated RNA Immunoprecipitation (MeRIP)-qPCR analysis of m^1^A level in *METTL3* mRNA in control and ALKBH1-depleted RKO cells. Statistical analysis was performed using an unpaired Two-tailed Student’s t test. (C) MeRIP-qPCR analysis of m^1^A level in *SMAD7* mRNA in control and ALKBH1-depleted RKO cells. Statistical analysis was performed using an unpaired Two-tailed Student’s t test. (D) MeRIP-qPCR analysis of m^6^A level in *SMAD7* mRNA in control and ALKBH1-depleted RKO cells. Statistical analysis was performed using an unpaired Two-tailed Student’s t test. (E) MeRIP-qPCR analysis of m^6^A level in *SMAD7* mRNA in control and METTL3-depleted RKO cells. Statistical analysis was performed using an unpaired Two-tailed Student’s t test. (F-H) RKO cells transfected with the indicated siRNAs or vectors were subjected to western blot analysis of the expression of SMAD7, METTL3 and ALKBH1. GAPDH, a loading control. (I-K) RKO cells co-transfected with the indicated siRNAs and vectors were subjected to western blot analysis with the indicated antibodies. GAPDH, a loading control. Quantitative data from three independent experiments are shown as the mean ± SD. **P* < 0.05; ****P* < 0.001; ns not significant.

**Fig. S13**

**
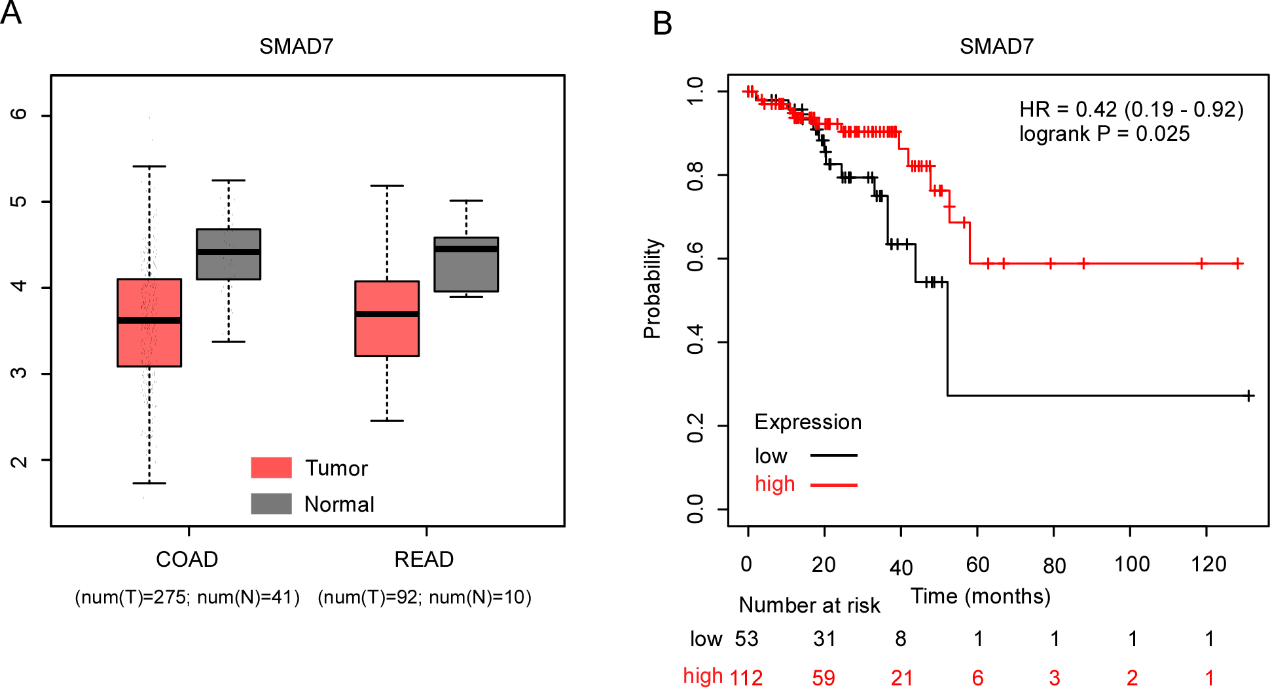
**

**Figure. S13.** **SMAD7 is downregulated in colorectal cancer (CRC) tissues and related to the poor prognosis in patients.** (A) Analyses of *SMAD7* mRNA levels of colon adenocarcinoma (COAD) and rectum adenocarcinoma (READ) tissues using Gene Expression Profiling Interactive Analysis (GEPIA). (B) Kaplan-Meier analysis of *SMAD7* mRNA expression in rectum adenocarcinoma from KM plotter database.

**Fig. S14**

**
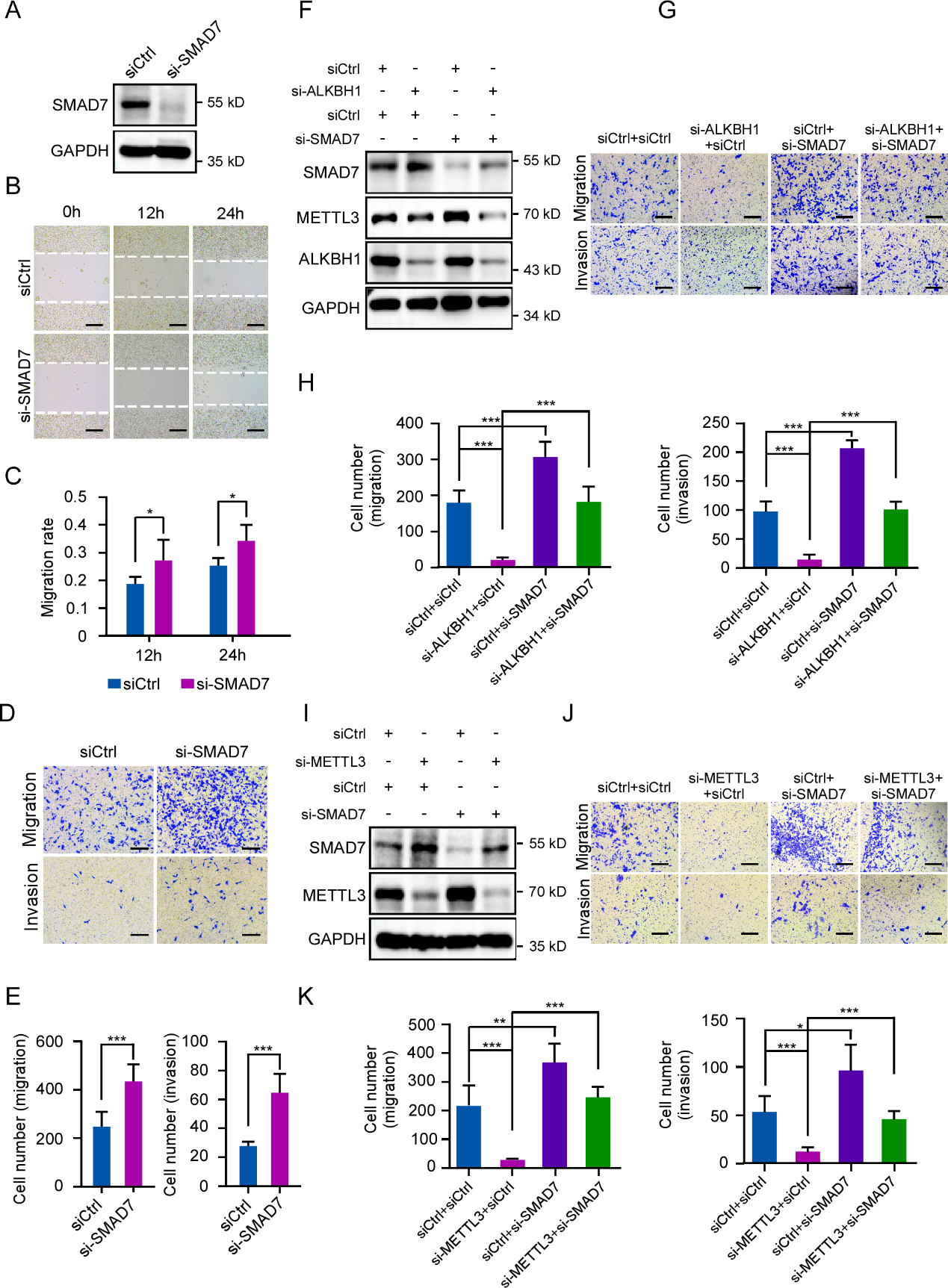
**

**Figure. S14. ALKBH1 boosts the invasiveness of RKO cells by downregulating SMAD7 expression.** RKO cells transfected with the indicated siRNAs and vectors were subjected to the following analyses: (A) RKO cells were transfected with control or SMAD7 siRNAs for 48 h and then subjected to western blot analysis with anti-SMAD7 antibody. GAPDH, a loading control. (B, C) The wound healing assays displayed cell migration at the different time points. Dashed lines indicate the wound edges. Scale bar, 200 µm. The distance between the two edge lines was measured by ImageJ software. Comparisons were analyzed by an unpaired Two-tailed Student’s t test. (D, E) Transwell migration and invasion assays revealed the cell migration and invasion abilities of control and SMAD7-depleted cells. Scale bar, 200 µm. Cells that moved to the undersides of the filters were counted. Comparisons were analyzed by an unpaired Two-tailed Student’s t test. (F) Western blotting analysis of the expression of SMAD7, METTL3 and ALKBH1. GAPDH was served as a loading control. (G, H) Transwell migration and invasion assays revealed the cell migration and invasion abilities. Scale bar, 200 µm. Cells that migrated to the undersides of the filters were counted. Comparisons were analyzed by One-way ANOVA. (I) Western blotting analysis of the expression of SMAD7 and METTL3. GAPDH was served as a loading control. (J, K) Transwell migration and invasion assays revealed the cell migration and invasion abilities. Scale bar, 200 µm. Cells that migrated to the undersides of the filters were counted. Comparisons were analyzed by One-way ANOVA. Quantitative data from three independent experiments are shown as the mean ± SD. **P* < 0.05; ***P* < 0.01; ****P* < 0.001.

**Fig. S15**


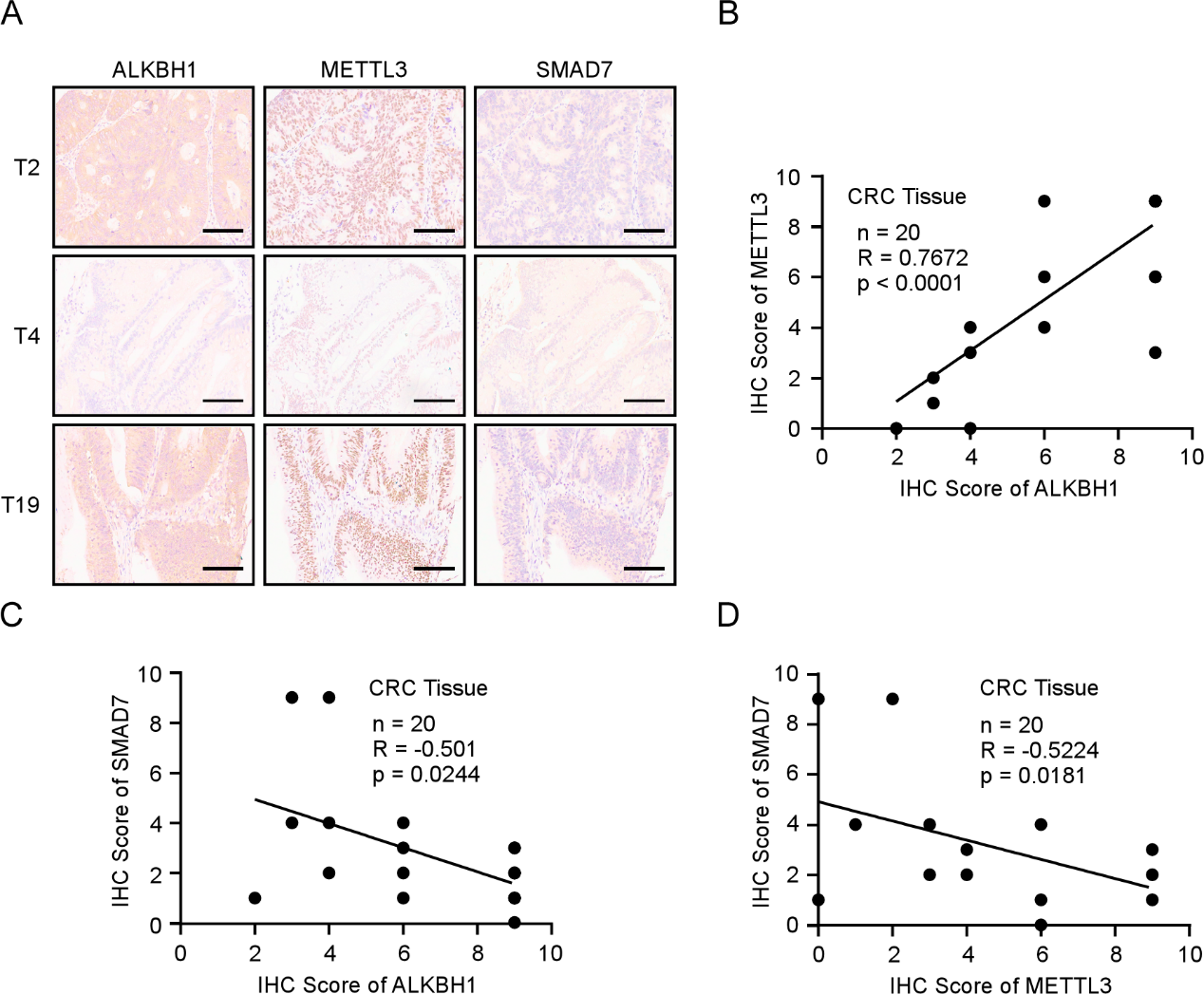


**Figure. S15.** **The clinical correlation between ALKBH1, METTL3 and SMAD7 expression in colorectal cancer (CRC) tissue samples.** (A)The expression levels of ALKBH1, METTL3 and SMAD7 were detected by immunochemistry analysis in 20 CRC tissues. Representative images in 3 tumor (T) tissues were shown. Scale bar, 100 µm. (B) The correlation between ALKBH1 and METTL3 protein levels in CRC tissues. (C) The correlation between ALKBH1 and SMAD7 protein levels in CRC tissues. (D) The correlation between METTL3 and SMAD7 protein levels in CRC tissues.
